# Type I Framework Complex: Photocontrolled Superoxide Anion Generator

**DOI:** 10.34133/research.1262

**Published:** 2026-04-27

**Authors:** Zehao Jing, Yingying Zhang, Yingnan Wu, Xiaoqiang Chen, Meizhen Yin, Mingle Li, Xiaojun Peng

**Affiliations:** ^1^ State Key Laboratory of Fine Chemicals, College of Materials Science and Engineering, Shenzhen University, Shenzhen 518071, China.; ^2^ State Key Laboratory of Chemical Resource Engineering, Beijing University of Chemical Technology, Beijing 100029, China.; ^3^ State Key Laboratory of Fine Chemicals, Frontiers Science Center for Smart Materials, Dalian University of Technology, Dalian 116024, China.

## Abstract

Photodynamic therapy (PDT) is a clinically approved therapeutic modality that uses photosensitizers (PSs) to generate reactive oxygen species (ROS) upon light irradiation, enabling disease treatment with minimal invasiveness and excellent spatiotemporal precision. Despite these advantages, conventional PDT is fundamentally constrained by the mismatch between its oxygen dependence and the intrinsically hypoxic tumor microenvironment, which markedly compromises therapeutic outcomes. In this context, type I PSs offer a promising solution because they can produce cytotoxic radicals through electron transfer pathways, thereby reducing dependence on oxygen (O_2_) and improving efficacy under hypoxic conditions. Organic framework materials have recently emerged as powerful and versatile platforms for constructing type I PSs, owing to their programmable structures, high porosity, and efficient photoinduced charge separation and electron transfer. Importantly, the modular nature of these frameworks enables rational tuning of both structural motifs and compositional building blocks, allowing systematic regulation of light absorption, redox properties, and ROS generation pathways to maximize type I PDT performance. Moreover, organic frameworks can simultaneously function as nanocarriers for therapeutics, facilitating co-delivery and synergistic combinations (e.g., chemotherapy, immunotherapy, or catalytic therapies) that may achieve more durable and comprehensive tumor control. However, current studies remain fragmented, and there is still a lack of an integrated and mechanistically grounded overview that connects framework design principles with type I ROS generation mechanisms and performance optimization strategies. To address this unmet need, this review provides a comprehensive summary of the design strategies, mechanistic insights, and recent progress in organic framework-based type I PSs. We first outline the fundamental principles of type I photochemistry and the key physical and chemical processes underlying type I PDT. We then highlight rational design and modulation strategies to enhance optical properties, promote charge separation, and strengthen oxygen independence. Next, we summarize representative in vivo/in vitro disease models to demonstrate emerging diagnostic and therapeutic applications. Finally, we discuss current challenges and future opportunities for clinical translation, offering practical guidance for the development of next-generation phototherapeutic agents based on these innovative framework systems.

## Introduction

Cancer is now a leading cause of premature mortality in most countries, and since the early 21st century, it has become a global public health challenge that imposes substantial long-term clinical and socioeconomic burdens on patients and healthcare systems. Photodynamic therapy (PDT) is a noninvasive photochemical treatment modality in which photosensitizers (PSs) convert light energy into chemical reactivity. Upon irradiation, PSs generate cytotoxic reactive oxygen species (ROS), giving PDT broad relevance in antitumor and antibacterial interventions [1,2]. Therapeutic efficacy requires the coordinated presence of 3 essential components: (a) efficient and ideally selective accumulation of PSs in the target tissue, (b) irradiation with a light source matched to the PS absorption profile, and (c) sufficient molecular oxygen (O_2_) in the local microenvironment [3]. Following photoactivation, excited PSs initiate ROS production, leading to rapid ROS accumulation and intense oxidative stress. This oxidative insult irreversibly damages critical biomolecules—including DNA, lipids, and proteins—thereby activating programmed cell death pathways and eliminating diseased cells [4].

**Fig. 1.**
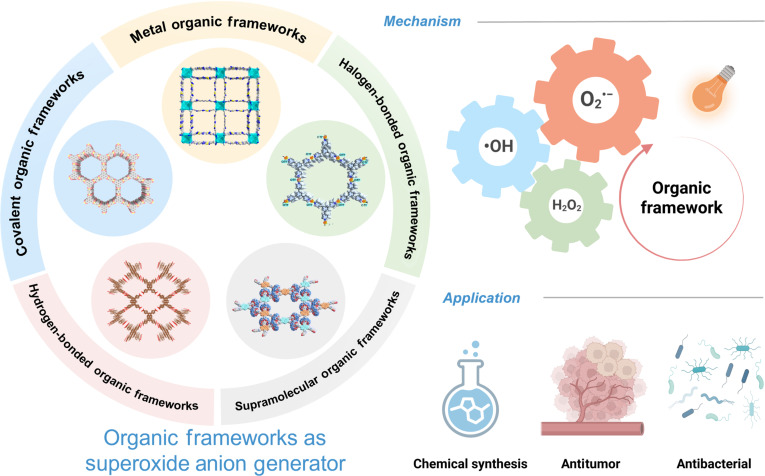
The mechanism of ROS generation in type I organic framework materials and their applications in chemical synthesis, antitumor, and antibacterial. Reproduced with permission of ref. [39], Copyright © 2021 American Chemical Society; reproduced with permission of ref. [147], Copyright © 2024 Wiley-VCH GmbH*.* Figure created with Biorender.com

According to ROS generation mechanisms, PDT is generally classified as type I or type II. In type II PDT, ROS production mainly proceeds via energy transfer (ET) from the excited PS to O_2_ to generate singlet oxygen (^1^O_2_). This pathway is common for many small-molecule PSs: Intersystem crossing (ISC) populates the triplet state, which subsequently transfers energy to O_2_ to form ^1^O_2_ and induce oxidative damage. However, type II PDT is strongly O_2_-dependent; its efficacy is markedly reduced in hypoxic settings such as solid tumors, where limited O_2_ becomes a fundamental bottleneck [5]. In contrast, type I PDT proceeds primarily via an electron transfer pathway. Upon photoexcitation, the PS interacts with surrounding substrates (e.g., cellular membranes or biomolecules) and/or O_2_ through electron donation or abstraction. This process generates radical species, including superoxide anion (O_2_^•−^), hydroxyl radicals (•OH), and hydrogen peroxide (H_2_O_2_), which collectively contribute to oxidative damage [6]. Compared with the strict O_2_ requirement of type II PDT, the electron transfer mechanism of type I systems exhibits reduced O_2_ dependence. The initial redox reactions can occur even under relatively low O_2_ levels, and subsequent radical chain processes can further amplify cytotoxic effects. This mechanistic distinction is particularly important in the context of solid tumors, where hypoxia is a hallmark feature resulting from abnormal vasculature and rapid cellular proliferation. By leveraging an electron transfer-dominated ROS generation pathway, type I PDT provides a strategically advantageous approach for hypoxic tumor treatment [7] (Fig. 1).

Type I PSs include small-molecule agents and inorganic (or hybrid) nanomaterials [8], which can follow distinct routes for O_2_^•−^ generation and downstream ROS evolution [9,10]. Small-molecule PSs are structurally well-defined, synthetically accessible, and readily functionalized to improve photosensitivity or introduce targeting motifs [11]. Nevertheless, a key challenge is to simultaneously optimize fluorescence output, electron transfer efficiency, and ET behavior, because these photophysical pathways often compete with one another. Consequently, the development of type I small-molecule PSs still relies heavily on empirical screening, and broadly applicable structure–activity relationships and rational design principles remain insufficiently established. By comparison, inorganic (hybrid) nanomaterials, particularly organic framework-based materials, offer a compelling and highly designable alternative for type I PDT. These frameworks feature programmable architectures, high crystallinity and stability, and exceptional modularity in both composition and function [12]. Their intrinsic porosity provides abundant accessible active sites and multiple transport channels, facilitating rapid migration and utilization of photogenerated charges. This can suppress bulk electron hole recombination and thereby enhance charge separation and electron transfer efficiency, which are the key determinants of type I ROS production [13]. Importantly, the precise and modular tunability of organic frameworks enables a more systematic, mechanism-guided route to type I PS design, offering opportunities to regulate light harvesting, redox properties, and electron transfer pathways at the structural level rather than by trial-and-error screening.

Building on these advantages, this review summarizes recent progress in organic framework-based type I PSs, with an emphasis on structure–mechanism–function relationships that govern type I ROS generation and therapeutic performance across diverse pathological conditions (e.g., cancer and inflammation). We first introduce the major classes and physicochemical features of type I ROS, with particular attention to their interactions with biomacromolecules in vivo (including proteins, DNA, lipids, and amino acids) [14]. We then discuss performance enhancement strategies for different categories of organic framework materials from a structural design perspective, highlighting how rational regulation of framework motifs, electronic structures, and charge transfer pathways can improve O_2_ tolerance and type I ROS yields. Next, we present representative diagnostic and therapeutic applications, spanning in vivo disease models and in vivo/in vitro tissue-based evaluations. Finally, we summarize remaining challenges and outline future directions toward clinically translatable, precision phototherapy, aiming to guide the development of next-generation type I phototherapeutic agents in this rapidly evolving field.

## ROS—a Key Role in PDT

The therapeutic efficacy of PDT originates from its ability to trigger light-driven redox reactions that generate ROS. Therefore, a mechanistic understanding of ROS formation and evolution is essential for the rational development of efficient and precise PDT systems. In this section, we systematically dissect the key reactive intermediates and core photochemical processes underlying type I PDT to establish a clear framework that links PS structure, charge transfer pathways, and ROS output.

### ROS generation mechanism

To date, type I PSs can be broadly classified into 2 representative categories: (a) ordered hybrid/inorganic nanomaterials, typified by organic framework-based materials, and (b) organic molecular dyes. The hallmark of type I PDT is electron transfer-dominated photochemistry, in which photogenerated charges on the PS participate in redox reactions with O_2_ and/or surrounding substrates to yield radical species (e.g., O_2_^•−^ and •OH) [6]. Importantly, the dominant electron transfer mode differs, reflecting their distinct electronic structures and photophysical behaviors.

**Fig. 2.**
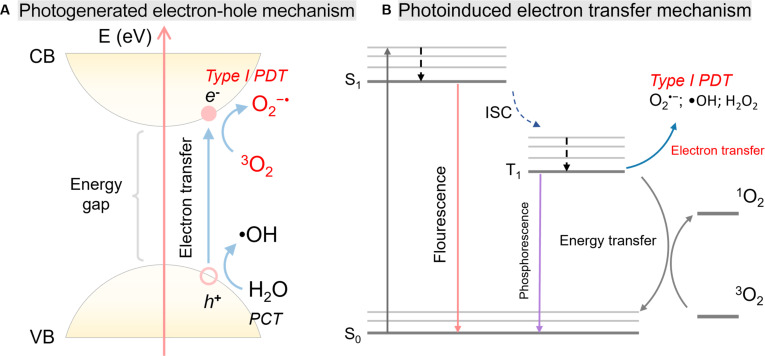
(A) Schematic diagram of the typical excitation process in organic frameworks, illustrating the transition of electrons from the VB to the CB upon photoirradiation, followed by type I PDT processes. Reproduced with permission of ref. [8], Copyright © 2025, Royal Society of Chemistry. (B) Type I PDT mechanism in organic molecules and Jablonski energy diagram of photochemistry in PDT. Reproduced with permission of ref. [8], Copyright © 2025, Royal Society of Chemistry.

Organic framework-based type I PSs predominantly operate through a photogenerated electron–hole mechanism (Fig. 2A) [15]. In this context, the bandgap (Eg) becomes a decisive design parameter because it governs light harvesting and charge excitation. Upon irradiation, the framework absorbs photons; when the photon energy is equal to or greater than Eg, electrons are promoted from the valence band (VB) to the conduction band (CB), leaving behind holes in the VB that are often delocalized over the ligand motifs [16]. The resulting spatially separated charges enable 2 coupled redox half-reactions: Electrons in the CB reduce O_2_ to generate O_2_^•−^, while holes in the VB oxidize H_2_O (or OH^−^) to form •OH [17]. Notably, hole-driven water oxidation can proceed without requiring O_2_, offering a partially O_2_-independent route to cytotoxic ROS generation. The produced radicals, owing to their high oxidative potential, can damage membrane structures and intracellular biomacromolecules, ultimately initiating irreversible cellular injury and cell death.

By contrast, small-molecule type I PSs typically generate radicals via photoinduced electron transfer (PET) from molecular excited states (Fig. 2B) [8]. After photon absorption, the PS is promoted to a short-lived singlet excited state (^1^PS*), which may return to the ground state (S_0_) through radiative (fluorescence) or nonradiative decay pathways. Alternatively, ^1^PS* can undergo ISC to populate a longer-lived triplet state (^3^PS*), which is generally responsible for subsequent type I reactivity [14]. The ^3^PS* state can engage in single-electron transfer (SET) with O_2_ to yield O_2_^•−^, and related downstream pathways can further produce additional ROS. In aqueous environments, oxidative pathways associated with hole transfer and secondary reactions may also contribute to •OH formation, complementing O_2_^•−^-based radical chemistry [7,8].

A mechanistic view of type I ROS generation, centered on photoinduced electron transfer and the distinct reactivities of O_2_^•−^, •OH, and H_2_O_2_, provides a rigorous foundation for evaluating and designing next-generation PSs. To act as an effective type I PS for PDT, a material must not only form these radicals upon irradiation but also do so efficiently under biologically relevant conditions, where competing quenchers, limited O_2_ availability, and complex interfacial environments can suppress reactivity [18]. Meeting this requirement demands deliberate control over charge separation efficiency, electron transfer kinetics, and interfacial redox chemistry so that photogenerated electrons and holes are funneled into productive ROS-forming pathways rather than dissipative recombination. In the following section, we highlight how organic framework materials—with programmable composition and topology, tunable electronic structure, and ordered porous interfaces—can be rationally engineered to satisfy these constraints. We emphasize design-relevant photophysical descriptors (e.g., band-edge alignment, carrier lifetimes, and interfacial charge transfer rates) that directly determine the feasibility and efficiency of electron transfer reactions responsible for type I ROS, thereby linking fundamental photochemistry to actionable materials design rules.

### Physical and chemical properties of type I ROS

**Fig. 3.**
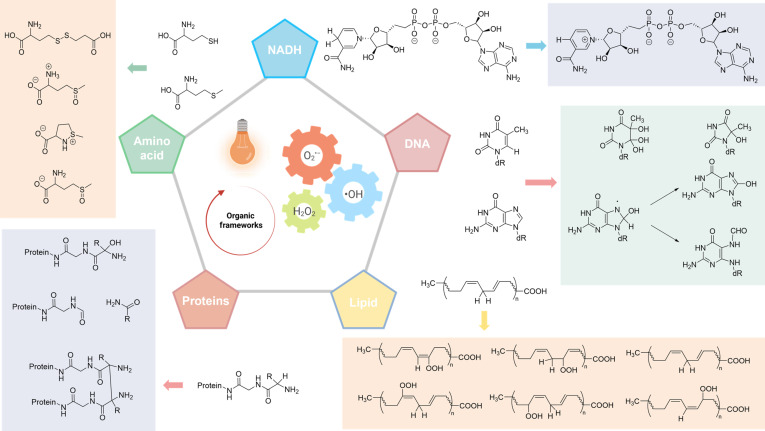
The oxidation pathways of biomolecules mediated by ROS generated in type I PDT processes. This diagram illustrates key oxidative reactions induced by ROS, including •OH, O_2_^•−^, H_2_O_2_, and associated cascade reactions. Reproduced with permission of ref. [8], Copyright © 2025, Royal Society of Chemistry.

ROS comprise a heterogeneous family of O_2_-derived oxidants with high redox activity in both biological and environmental systems. At supraphysiological levels, ROS induce largely nonselective oxidative modifications of biomacromolecules, including protein carbonylation, lipid peroxidation, nucleic acid strand scission, and carbohydrate oxidation, often generating cytotoxic secondary products. In contrast, at physiological concentrations, ROS serve as indispensable signaling mediators that regulate signal transduction cascades, cell cycle progression, and redox homeostasis [19]. During PDT, PS-driven ROS production can overwhelm endogenous antioxidant defenses, thereby triggering oxidative stress and extensive macromolecular injury. This damage, manifested as DNA fragmentation, membrane disruption, and protein denaturation, ultimately activates cell death programs such as mitochondrial apoptosis, ferroptosis, and/or necrosis (Fig. 3). In type I PDT, 3 ROS are especially pivotal as reactive intermediates and effectors: O_2_^•−^, •OH, and H_2_O_2_. Below, we summarize their key photophysical /photochemical features and biological consequences, emphasizing why controlling ROS identity and flux is central to mechanism-guided type I PDT design.

#### Superoxide radical (O_2_^•−^)

O_2_^•−^ is a paramagnetic, short-lived species produced by single-electron reduction of ground-state triplet oxygen (^3^O_2_). It exhibits dual radical-anionic character, with reactivity arising from an unpaired electron occupying an antibonding π* orbital [20]. Its fate is strongly microenvironment-dependent (e.g., pH and ionic strength), with a physiological half-life typically <50 ms and a diffusion radius on the submicrometer scale.

Electron delocalization reduces the formal charge per oxygen atom (≈ −0.5), elongating the O–O bond (~1.34 Å versus ~1.21 Å in O_2_) while preserving partial double-bond character [21]. The relatively modest reduction potential of the O_2_/O_2_^•−^ couple [~+0.16 V versus NHE (normal hydrogen electrode)] and the low p*K*_a_ (where *K*_a_ is the acid dissociation constant) of its conjugate acid (p*K*_a_ ≈ 4.8) mean that O_2_^•−^ remains largely deprotonated at physiological pH [8]. Under acidic conditions, however, protonation yields the hydroperoxyl radical (•HO_2_), which can participate in downstream redox cascades that generate H_2_O_2_ and, indirectly, •OH. Key downstream reactions include O_2_^•−^ dismutation [spontaneous or SOD (superoxide dismutase)-catalyzed] and Fe-mediated redox cycling (Fenton/Haber–Weiss chemistry):

$$O_{2}^{•-}+H^{+}\to H_{2}O_{2}+O_{2}\;\;$$ (1)

$$O_{2}+{\mathrm{Fe}}^{2+}\to {\mathrm{Fe}}^{3+}+•\mathrm{OH}+{\mathrm{OH}}^{-}\;$$ (2)

$$O_{2}^{•-}+{\mathrm{Fe}}^{3+}\to {\mathrm{Fe}}^{2+}+O_{2}\;\;$$ (3)

$$O_{2}^{•-}+H_{2}O_{2}\to O_{2}+•\mathrm{OH}+{\mathrm{OH}}^{-}\;$$ (4)

In cells, mitochondrial electron transport is a major endogenous source of O_2_^•−^, whereas O_2_^•−^ dismutases and antioxidant networks tightly regulate its steady-state level to maintain redox balance. From a type I PDT perspective, O_2_^•−^ is often a “gateway ROS”—less reactive than •OH but capable of feeding into H_2_O_2_/•OH-generating cascades, thereby amplifying oxidative injury.

#### Hydroxyl radicals (•OH)

•OH is among the most reactive ROS known and is frequently the terminal cytotoxic effector in type I PDT. It can arise from Fenton/Fenton-like reactions (e.g., Fe^2+^/Cu^+^-catalyzed conversion of H_2_O_2_) and, in certain photoredox systems, from direct oxidation of H_2_O/OH^−^ by highly oxidizing holes. The •OH/H_2_O redox couple has an exceptionally high potential (commonly cited around +2.3 V versus NHE), enabling •OH to oxidize or abstract hydrogen from most nearby biomolecules with diffusion-limited rate constants (~10^9^ to 10^10^ M^−1^ s^−1^) [22]. Because of this extreme reactivity, •OH is consumed essentially at its site of formation, leading to ultralow steady-state concentrations (often pM-range) and highly localized damage. Biologically, •OH attacks nearly all classes of biomolecules, including oxidative base modification and strand breaks in DNA, rapid lipid peroxidation (which can proceed without requiring additional O_2_ once initiated), and irreversible oxidation/fragmentation of proteins [23]. Importantly, H_2_O_2_ is comparatively mild until it is converted into •OH, underscoring why many type I PDT strategies aim to boost •OH generation rather than merely increasing total ROS.

#### Hydrogen peroxide (H_2_O_2_)

H_2_O_2_ is the 2-electron reduction product of O_2_ and exists predominantly in its undissociated form under physiological conditions (p*K*_a1_ ≈ 11.8) [24]. Unlike •OH, H_2_O_2_ is relatively longer-lived and can diffuse farther, making it both a signaling molecule and a reservoir that can be converted into more aggressive oxidants. A defining chemical feature of H_2_O_2_ is its reactivity toward thiols, allowing it to modulate sulfur-containing biomolecules such as glutathione and cysteine residues. The oxygen atoms in H_2_O_2_ have an oxidation state of −1, giving H_2_O_2_ both oxidizing and reducing character; its redox activity is mediated by O–O bond cleavage and H–O bond transformations [25]. Transition-metal ions (e.g., Fe, Co, and Mn) can efficiently activate H_2_O_2_ to yield •OH, a property widely leveraged to intensify oxidative damage via Fenton/Fenton-like pathways. In type I PDT, H_2_O_2_ typically arises from ROS cascade chemistry rather than direct excitation of O_2_ [26]. For small-molecule PSs, O_2_^•−^ generated by SET can undergo dismutation (often SOD-mediated) to form H_2_O_2_. For organic framework-based PSs, a distinctive advantage is mechanistic plurality: H_2_O_2_ can be produced through multiple photoredox routes, including (a) O_2_ reduction reaction (ORR) driven by photogenerated electrons and (b) water oxidation-linked pathways driven by photogenerated holes. In a stepwise single-electron ORR sequence, electrons reduce O_2_ to O_2_^•−^, followed by further electron/proton-coupled steps that yield H_2_O_2_. In parallel, hole-mediated oxidation of H_2_O can generate •OH (and H^+^), and radical recombination/secondary reactions can also contribute to H_2_O_2_ formation. In some systems, more direct multi-electron ORR channels have been proposed, providing an additional lever to tune H_2_O_2_ output by engineering interfacial active sites and charge transfer kinetics. Endogenously, cellular H_2_O_2_ is tightly regulated: O_2_^•−^ dismutase produces H_2_O_2_ from O_2_^•−^, while catalase (CAT), peroxidases, and other peroxide-metabolizing enzymes govern its clearance and compartmentalization [27]. CAT is particularly important because it decomposes H_2_O_2_ into H_2_O and O_2_, which can, in principle, feed back into O_2_-dependent steps and partially reshape local O_2_ availability [28]. Ascorbate peroxidase and related peroxidases decompose H_2_O_2_ via distinct catalytic cycles, producing water and reactive radical intermediates [29].

### Classification of type I and type II PDT

It is important to note that although type I PDT is generally less O_2_-dependent than type II PDT, it is not truly O_2_-independent. The initial electron transfer from the PSs to O_2_ can still proceed under hypoxic conditions, producing O_2_^•−^. Nevertheless, subsequent radical reactions—and ultimately the therapeutic outcome—may remain constrained by the local O_2_ level, because O_2_ can participate in (or modulate) downstream redox pathways and reactive species propagation. For this reason, “hypoxia-tolerant” is a more precise description of type I PDT than “O_2_-independent”. To further reduce residual O_2_ dependence, recent innovative design strategies have focused on microenvironment engineering and O_2_ management, including (a) incorporating O_2_-recycling modules, such as CAT-mimetic frameworks that decompose endogenous H_2_O_2_ to regenerate O_2_ [30], and (b) integrating in situ O_2_-generating components to locally replenish oxygen during irradiation [31]. These approaches move beyond simply selecting type I PSs and represent a more active, systems-level route toward robust PDT in hypoxic tumors.

Although type I and type II photodynamic pathways are mechanistically distinct, they are not mutually exclusive [32]. In practice, many PSs can engage both routes from the excited state—producing ^1^O_2_ via ET while also generating radical species (e.g., O_2_^•−^ and •OH) through electron transfer. This raises a key question: How should such “hybrid” PSs be classified? The answer is not determined by whether multiple ROS can be detected, but by identifying which pathway dominates under biologically relevant conditions.

Following the recent guideline proposed by Li et al. [8], classification should be based on the primary mechanism responsible for therapeutic efficacy. If a PS mainly produces radicals through electron transfer (O_2_^•−^, •OH)—even if a small amount of ^1^O_2_ is detectable—it should be regarded as a type I PS. Conversely, if ET to O_2_ leading to ^1^O_2_ is the major route, the PS should be categorized as type II. Materials for which both pathways contribute substantially under relevant conditions are commonly described as dual-mode or type I/II PSs. Importantly, such systems are particularly attractive for treating solid tumors with heterogeneous oxygenation, because they can potentially maintain cytotoxicity in both normoxic and hypoxic regions—an innovation-relevant advantage for improving robustness in vivo.

In the following sections, we apply this mechanism-based classification framework to evaluate organic framework PSs. For materials capable of generating both ^1^O_2_ and O_2_^•−^, we explicitly highlight their dual-mode potential and discuss how their dominant ROS pathway may shift under hypoxic versus normoxic conditions. This approach ensures that our analysis is not merely descriptive, but mechanistically grounded, providing readers with a clear understanding of the therapeutic strengths, limitations, and translational implications of each material class.

## Framework Complexes for Superoxide Anion Generators

Building on these mechanistic principles, we then focus on the material platforms capable of implementing type I photochemistry in practice. Organic frameworks—including metal–organic frameworks (MOFs), covalent organic frameworks (COFs), hydrogen-bonded organic frameworks (HOFs), supramolecular organic frameworks (SOFs), and halogen-bonded organic frameworks (XOFs)—are emerging as particularly compelling candidates because they uniquely combine the following: (a) structural programmability, enabling precise modulation of light harvesting and redox potentials; (b) high porosity, promoting mass transport while exposing abundant, addressable reactive sites; (c) architecture-enabled charge separation, arising from ordered donor–acceptor (D–A) arrangements and/or node–linker electronic coupling; and (d) built-in multifunctionality, allowing integration with complementary therapeutic mechanisms. In this section, we critically assess each framework class through the lens of type I photochemistry, with particular attention to how structural and electronic features dictate electron transfer pathways, ROS selectivity, and overall PDT-relevant performance. Where evidence is available, we also discuss stability, biocompatibility, and in vivo behavior—translation-defining parameters that are frequently underreported in photocatalysis-focused studies, yet essential for identifying clinically realistic type I PS candidates. Type I photodynamic activity in organic framework-based PSs can be viewed as a coupled sequence of light harvesting, photoinduced electron hole generation and separation, charge migration, and interfacial electron transfer to O_2_ to yield radical ROS (for example, O_2_^•−^) [33]. Accordingly, strategies to enhance type I PDT performance converge on 2 core design axes: (a) Eg and band edge engineering to optimize light absorption and thermodynamic driving forces for charge separation and redox reactions, and (b) charge-transport engineering to accelerate carrier migration, suppress recombination, and prolong the lifetime of reactive charges. Within the first axis, a widely adopted approach is to introduce functional fragments—such as photoactive motifs, redox-active sites, or tailored secondary building units (SBUs)—to tune the intrinsic electronic structure of the parent framework [34]. In parallel, rational band alignment can be designed to match targeted redox couples, because the energetic cost of charge separation scales with Eg, whereas the oxidative and reductive capabilities are dictated by the VB and CB positions, respectively [35]. The second axis focuses on facilitating the migration of photogenerated electrons and holes through the framework, thereby minimizing electron–hole recombination and extending carrier lifetimes—parameters that are tightly coupled to type I ROS yields and photocatalytic efficiency.

**Fig. 4.**
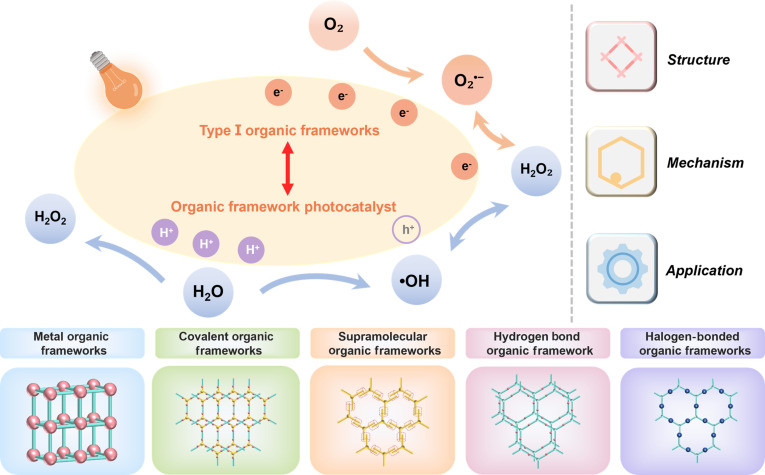
Organic framework photocatalysts with type I photodynamic potential, including MOF, COF, SOF, HOF, and XOF. All of them rely on the photogenerated electron–hole mechanism.

Although organic frameworks have been most extensively explored in photocatalysis, these same photoredox principles make them highly attractive as programmable type I PS platforms rather than merely passive carriers. Indeed, many frameworks already show strong photoinduced O_2_^•−^ generation (Fig. 4), underscoring their potential for type I PDT. In the following section, we highlight organic frameworks with type I PDT relevance and summarize key design/modification strategies.

To systematically and transparently evaluate organic frameworks as type I PSs for PDT, we propose a multi-parameter, translationally oriented benchmarking framework that unifies core photochemical performance with biological feasibility and clinical practicality. This framework considers: (a) type I ROS generation efficiency (e.g., quantum yields under biologically relevant conditions, including aqueous media and low O_2_ tension), (b) band structure and charge dynamics (bandgap energy, VB/CB positions, charge separation lifetime, and carrier mobility), (c) physiological stability [resistance to hydrolysis, aggregation, and enzymatic degradation in phosphate-buffered saline (PBS), serum, or cell culture media over relevant timescales], and (d) biocompatibility (dark cytotoxicity, hemocompatibility, and immunogenicity assessed by standard in vitro assays). Leveraging this innovation-enabling, cross-platform evaluation lens, we critically analyze MOFs, COFs, HOFs, SOFs, and XOFs in the following sections; where quantitative evidence remains limited, we explicitly flag these gaps as high-priority targets for future research to accelerate rational materials optimization and clinical translation.

### Metal–organic frameworks

MOFs are a class of porous crystalline materials assembled from metal ions or metal-oxo clusters and multitopic organic linkers via coordination bonding. By integrating inorganic nodes (structural rigidity and well-defined electronic states) with organic ligands (chemical diversity and functional tunability), MOFs offer an unusual combination of robustness and modularity. The directional nature of metal ligand coordination generates periodic lattices with well-defined pores and cavities, while overall stability is governed by coordination interactions whose strengths typically fall between covalent and noncovalent bonding [36]. Catalytically active sites in MOFs can arise from coordinatively unsaturated metal nodes, functionalized linkers, encapsulated guest species, and even structural defects, which often act as reactive “hot spots”. Owing to their compositional diversity (including access to a wide range of metal centers), high surface areas, large and tunable pore apertures, and programmable architectures, MOFs have become outstanding platforms for photocatalysis. Their porous networks not only expose abundant accessible sites but also promote reactant adsorption and mass transport—2 key determinants of catalytic efficiency. Crucially, the designability of both metal nodes and organic linkers enables systematic optimization: Rational selection and pairing of these components can precisely tailor light absorption, band-edge positions, and charge transfer pathways, thereby predictably tuning photocatalytic performance [37]. Together, these attributes position MOFs as a highly versatile and increasingly mechanism-engineerable materials platform for advanced photoredox applications.

#### Zn-MOF

**Fig. 5.**
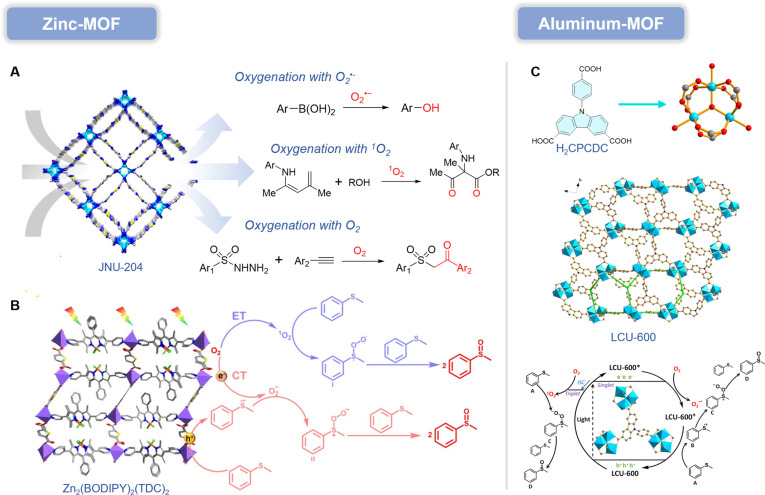
(A) Crystal structure of JNU-204 and proposed reaction mechanisms for the 3 photocatalytic oxidation reactions in the presence of JNU-204. Reproduced with permission of ref. [39], Copyright © 2021 American Chemical Society. (B) Structure of Zn_2_(BODIPY)_2_(TDC)_2_ and proposed mechanism for the photocatalytic hydroxylation for Zn_2_(BODIPY)_2_(TDC)_2_. Reproduced with permission of ref. [40], Copyright © 2024, Royal Society of Chemistry. (C) Structure of LCU-600 and proposed mechanism of photo-oxidation reaction of sulfides by LCU-600 in air atmosphere. Reproduced with permission of ref. [43], Copyright © 2024, American Chemical Society.

MOFs were first widely investigated as adsorbents for environmental pollutants; their high porosity and dispersed active sites also render them effective heterogeneous photocatalysts for oxidative transformations [38]. A key advantage over conventional semiconductors is that MOFs can be optimized through molecular-level design of both metal clusters and linkers, providing a uniquely precise handle to regulate photophysics and photochemistry. Li and colleagues [39] reported a novel pyrazole–benzothiadiazole–pyrazole PS integrated into a MOF (JNU-204) for photocatalytic aerobic oxidation (Fig. 5A). This PS features a D–A–D conjugated π-system enabling rapid charge separation. Its incorporation into the MOF yielded JNU-204, exhibiting exceptional photocatalytic performance. Under visible-light irradiation, photogenerated electrons in JNU-204 are excited from the π-system of the D–A–D structure to the LUMO (lowest unoccupied molecular orbital) energy level. These electrons subsequently transfer to O_2_ molecules via SET, generating O_2_^•−^. In the photocatalytic hydroxylation of arylboronic acids, O_2_^•−^ is initially generated via a SET process. Subsequently, the arylboronic acid reacts and abstracts a hydrogen atom from the triethylammonium radical cation. Finally, the rearrangement of the aryl group followed by hydrolysis yields the phenolic product. Notably, the presence of excess Et_3_N prevents over-oxidation to quinones. In the photocatalytic oxidation of enamines, ^1^O_2_ is initially generated through ET from photoexcited JNU-204. Enamines then react with ^1^O_2_ to form an organic peroxide intermediate. After dehydration, a 1,2-acyl migration driven by alcohol attack on the carbonyl carbon completes the construction of α-aminocarbonyl. In the photocatalytic oxy-sulfonylation of alkynes, the sulfonyl radical is generated through a sequence of oxidative reactions under visible-light irradiation. Subsequent addition to the alkyne produces a vinyl radical intermediate, which then traps molecular O_2_ to form a C–O bond. A subsequent SET process, protonation, and reduction furnish the final β-ketosulfone product. Concurrent energy and charge transfer mechanisms have been identified as critical factors in efficient photocatalytic oxidation of organic compounds.

Since it generates highly reactive intermediates through photon energy—intermediates difficult to access via conventional means—photoredox catalysis has emerged as a potent strategy for organic synthesis under environmentally benign conditions. Selective oxidation of organic compounds proves particularly valuable for synthesizing fine chemicals and pharmaceuticals. Utilizing pyridine-functionalized dipyrromethene (BODIPY) and 2,5-thiophenedicarboxylic acid (H_2_TDC), Wen and colleagues [40] synthesized a novel MOF [Zn_2_(BODIPY)_2_(TDC)_2_] through a one-pot solvothermal method. In the presence of H_2_TDC, BODIPY functions as both a light-harvesting antenna and a linker, exhibiting exceptional visible-light responsiveness and enabling effective photocatalytic oxidation of sulfides and arylboronic acids at ambient temperature (Fig. 5B). Zn_2_(BODIPY)_2_(TDC)_2_ maintains over 90% catalytic efficiency after 6 consecutive cycles, attributed to its outstanding visible-light absorption, stable crystalline framework, and efficient charge/ET processes. In the photocatalytic oxidation of sulfides, O_2_^•−^ and ^1^O_2_ radicals are both ROS, and sulfide radical cations may participate in the photooxidation process. Possible reaction mechanisms for sulfide photooxidation include a CT (charge transfer) pathway mediated by O_2_^•−^. In the ET pathway driven by blue light, O_2_ is converted to ^1^O_2_ through ISC between ^1^O_2_ and ^3^O_2_. Then, thioanisole reacts with ^1^O_2_ to generate a persulfoxide intermediate, and the persulfoxide intermediate reacts with thioanisole to produce 2 equivalents of sulfoxide product. In the CT pathway, charge separation occurs inside compound 1. Thioanisole is oxidized by photogenerated holes to the corresponding sulfide radical cation, while O_2_ is reduced by photogenerated electrons to O_2_^•−^. Subsequently, O_2_^•−^ reacts with the sulfide radical cation to form persulfoxide intermediate II, which is ultimately converted to the sulfoxide product. Therefore, the combination of ^1^O_2_ and O_2_^•−^ is the reason compound 1 possesses excellent activity.

Despite their strong photocatalytic performance and high O_2_^•−^ generation efficiency, the PDT potential of these Zn-MOFs remains largely underexplored, leaving a clear opportunity for translation-oriented innovation. A central barrier is physiological stability: Zn-based MOFs can be prone to hydrolysis in aqueous environments because Zn–carboxylate coordination bonds are relatively labile. For example, although JNU-204 was reported to be stable in the organic solvents used for photocatalysis, its structural integrity in PBS or cell culture media has not been demonstrated. Equally important, biocompatibility evidence is currently insufficient—key evaluations such as in vitro cytotoxicity and hemocompatibility testing have not yet been reported. Moving forward, systematic studies should quantify degradation kinetics in physiological buffers, identify and evaluate potentially harmful degradation products (e.g., released linkers and Zn-containing species), and establish dark toxicity and biosafety profiles before progressing to in vivo validation. In parallel, materials engineering strategies—such as PEGylation, polymer coatings, or protective core–shell encapsulation—may be required to improve aqueous stability, minimize nonspecific interactions, and ultimately enable clinically relevant PDT performance.

#### Al-MOFs

Aluminum-based MOFs (Al-MOFs) have garnered particular attention due to the high terrestrial abundance of aluminum. Furthermore, the high valence of Al^3+^ ensures exceptional thermal and chemical stability in Al-MOFs, which is critical for their durability and recyclability as catalysts [41]. Carbazole and its derivatives constitute a class of photoactive molecules with strong electron-donating capabilities, having been developed as photocatalysts for various photo-promoted organic reactions [42]. For instance, Su and colleagues [43] reported a visible-light-active carbazole-based catalyst with an exceptionally low excited-state oxidation potential, enabling the reduction of even deactivated chloroarenes.

Al-MOFs have emerged as ideal catalytic platforms owing to their high porosity, well-defined active sites, and designable pore architectures. Notably, the d^0^ electronic configuration of Al^3+^ helps prevent excited-state quenching while facilitating the integration of photoactive carbazole moieties into Al-MOFs. A robust, porous, and photoactive Al-MOF (LCU-600) was synthesized through the assembly of in situ-formed [Al_3_O(CO_2_)_6_] trinuclear building units and the carbazole-based tritopic ligand H_3_CPCDC [9-(4-carboxyphenyl) carbazole-3,6-dicarboxylic acid]. As it is shown in Fig. 5C, under illumination, charge separation within the LCU-600 framework generates free holes and electrons. Photogenerated holes oxidize methyl phenyl sulfide to its radical cation, while electrons reduce O_2_ to O_2_^•−^ via SET. The resulting sulfide radical cation reacts with O_2_^•−^ to form a persulfoxide molecule intermediate, which subsequently reacts with another sulfide to yield the corresponding sulfoxide. Alternatively, triplet-state electrons from the MOF’s LUMO undergo ET to convert O_2_ into ^1^O_2_, which reacts with MPS (methyl phenylsulfide) to generate an intermediate, ultimately producing sulfoxide. The presence of Al^3+^ endows LCU-600 with exceptional aqueous stability and permanent porosity toward N_2_ and CO_2_ adsorption. LCU-600 exhibits promising photoactivity and, benefiting from the high charge density of Al^3+^ and the formation of robust trinuclear [Al_3_O(CO_2_)_6_] SBUs, shows excellent hydrolytic stability and strong resistance to common organic solvents. This water stability is particularly attractive for biomedical use, where structural integrity in aqueous and saline environments is a prerequisite for reliable PDT performance. However, direct evidence supporting its biosafety is still lacking. Although aluminum-based frameworks are often regarded as relatively low-toxicity materials, the cell type-specific cytotoxicity of LCU-600 has not been validated in mammalian systems, and its behavior in complex biological milieus remains uncharacterized. From an innovation standpoint, the exceptional aqueous stability of LCU-600 makes it a compelling platform MOF for translation-oriented engineering: Its robust Al-cluster scaffold could enable surface functionalization (e.g., PEGylation or zwitterionic coatings) to enhance colloidal stability and reduce nonspecific adsorption while offering a chemically resilient host for structure–property tuning (e.g., linker modulation or heteroatom/guest incorporation) to optimize type I charge transfer pathways under hypoxia. Together, these studies would transform LCU-600 from a chemically stable photocatalyst into a clinically actionable PDT candidate supported by quantitative biosafety and biointerface evidence.

#### Zr-MOFs

**Fig. 6.**
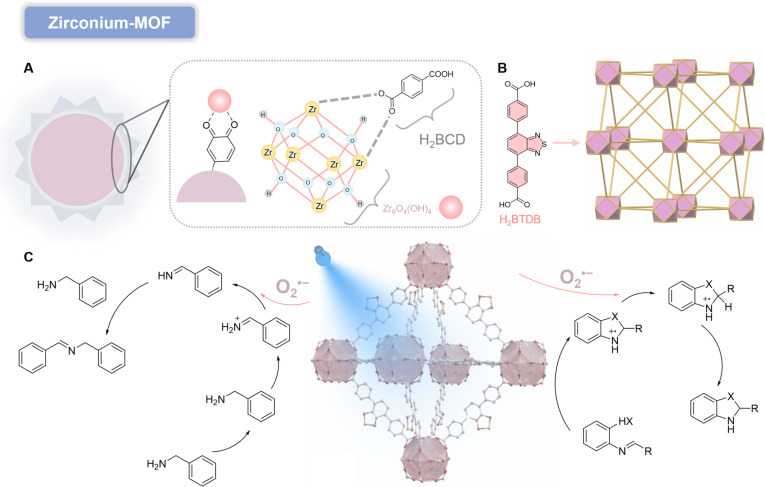
(A) Plausible mechanism of UiO-68-BTDB catalyzed the condensation cyclization reaction to synthesize benzimidazoles and benzothiazoles. Reproduced with permission of ref. [46], Copyright © 2020, American Chemical Society. (B) Schematic representation of UiO-68-NH_2_ and UiO-68-BT. Reproduced with permission of ref. [48], Copyright © 2024, Royal Society of Chemistry. (C) A possible mechanism for the blue light-driven selective aerobic oxidation of benzylamine by UiO-68-BT photocatalysis. Reproduced with permission of ref. [48], Copyright © 2024, Royal Society of Chemistry.

Zirconium-based MOFs (Zr-MOFs) have been widely employed as structurally robust catalysts for wastewater treatment due to their tunable micro/mesopore diameters, adjustable architectures, and large specific surface areas [44]. Recent studies focus on integrating electron acceptors [e.g., H_2_O_2_, persulfate (PS), and peroxymonosulfate (PMS)] to mitigate electron–hole recombination and enhance photocatalytic performance. These oxidants act as external electron scavengers, promoting •OH generation [45]. Salimi and colleagues [46] developed a bioinspired core/shell Zr-MOF (UiO-66@PDA) using polydopamine nanoparticles (PDA NPs) as templates for visible-LED (light-emitting diode) photocatalysis. The PDA@MOF hybrid combines PDA’s adhesive, hydrophilic, and redox-active properties with MOF crystallinity. The catechol-to-catecholquinone transition on PDA surfaces facilitates Zr^4+^chelation, enabling strong chemical bonding with organic linkers (H_2_BDC) and spontaneous MOF growth (Fig. 6A).

This nanostructure promotes O_2_ reduction to O₂^•−^ and •OH, achieving efficient methylene blue degradation under visible-LED irradiation. With H_2_O_2_ as an electron acceptor, the system achieves 99% methylene blue decolorization within 80 min, attributed to its core/shell architecture, high surface area, optimal Eg, and effective molecular sieving. Among photoactive compounds, benzothiadiazole derivatives with bicyclic electron-deficient frameworks exhibit unique optoelectronic properties due to their D–A–D design [47]. Zhao and colleagues [48] constructed a visible-light-active MOF (UiO-68-BTDB) using 4,4′-(benzo[c][1,2,5]thiadiazole-4,7-diyl) dibenzoic acid (H_2_BTDB) as a PS. The well-isolated benzothiadiazole chromophores in UiO-68-BTDB enable simultaneous generation of ^1^O_2_ and O_2_^•−^ (Fig. 6B). A proposed mechanism for benzimidazole/benzothiazole condensation involves (a) *o*-phenylenediamine/*o*-aminothiophenol and benzaldehyde dehydration to form Intermediate I; (b) charge separation under visible light; (c) hole-mediated oxidation of I to Intermediate II, which undergoes deprotonation and cyclization; (d) triplet-state electrons generating ^1^O_2_ via ET; and (e) ^1^O_2_-mediated oxidation of intermediates to final heterocyclic products. Concurrently, photogenerated electrons reduce O_2_ to O_2_^•−^, which participates in alternative reaction pathways. Modifications of organic linkers and metal nodes can extend MOF absorption from ultraviolet (UV) to visible and even infrared regions [49]. The permanent porosity and high surface area of MOFs facilitate rapid redox reactions between charge carriers (e^−^/H^+^) and reactants, effectively suppressing carrier recombination [50] (Fig. 6C). On this basis, Lang and colleagues [51] developed Zr-MOFs (UiO-68-NH_2_ and UiO-68-BT) using amino-TPDC (2′-amino-[1,1′:4′,1″-terphenyl]-4,4″-dicarboxylic acid) and H_2_BTDB ligands with Zr_6_O_4_(OH)_4_^12+^ clusters. Under blue light, UiO-68-BT generates e^−^/H^+^ pairs: O_2_ scavenges e^−^ to form O_2_^•−^, while H^+^ oxidizes x benzylamine to radical cations. O_2_^•−^-mediated oxidation then yields N-benzylbenzenediamine and NH_3_. Theoretical calculations confirm that electron-withdrawing benzothiadiazole units in UiO-68-BT significantly enhance charge separation and visible-light activity compared to UiO-68-NH_2_.

Zr-MOFs, particularly those derived from the UiO-66 platform, are highly regarded for their exceptional chemical and thermal stability, which arises from the robust Zr–O coordination bonds and the high connectivity of Zr_6_ clusters. This inherent stability translates into strong resistance against hydrolysis in physiological buffers, making them promising candidates for biological and medical applications. Several studies have assessed the biocompatibility of UiO-66 NPs, reporting low cytotoxicity (cell viability >80% at concentrations up to 100 μg/ml) in various cell lines, including HeLa and MCF-7, thus establishing their potential for safe application in vitro. However, the specific frameworks discussed here—UiO-68-BTDB and UiO-68-BT—have not yet been subjected to similar biocompatibility assessments. These frameworks differ from the prototypical UiO-66 in key aspects, including larger pore sizes and extended linkers, which could potentially alter their degradation behavior and biocompatibility. Furthermore, the incorporation of benzothiadiazole units, known for their optoelectronic properties, introduces an additional layer of complexity. While these units may enhance photocatalytic efficiency, their biological effects remain largely unexplored, raising concerns about the toxicity of the leached linkers. To address these gaps, future research should focus on several key areas: (a) evaluating the colloidal stability of these MOFs in serum-containing media, (b) investigating hemolysis potential, and (c) conducting acute toxicity studies in vivo to fully assess the biocompatibility and safety of these novel frameworks. Notably, the core/shell UiO-66@PDA structure developed by Salimi and colleagues represents an important innovation, as the PDA coating significantly enhances biocompatibility by reducing cytotoxicity and improving cellular uptake. This approach exemplifies how surface functionalization can be employed to optimize MOF performance for biomedical applications, creating a pathway for more efficient and safer PDT delivery systems.

#### Ni-MOF

**Fig. 7.**
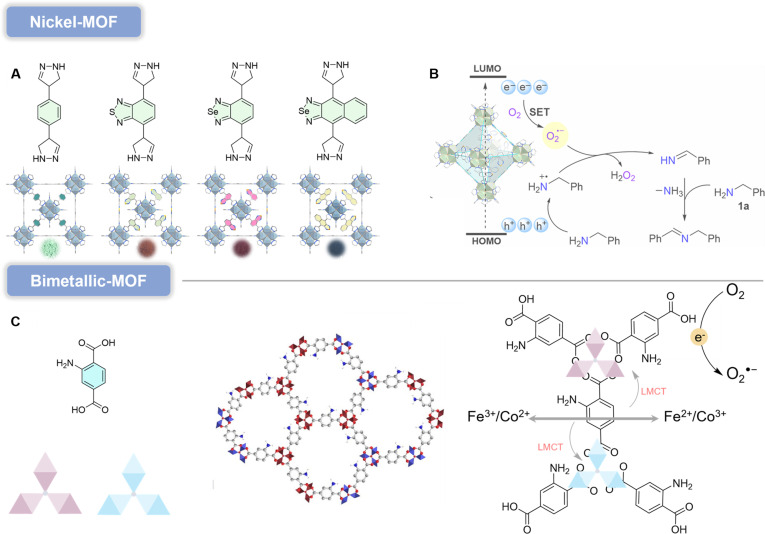
(A) Schematic illustration of linker engineering in the synthesis of ultra-stable Ni-MOFs. (B) Plausible reaction mechanisms of aerobic benzylamine oxidation with JNU-214 as a photocatalyst. Reproduced with permission of ref. [54], Copyright © 2023, American Chemical Society. (C) Schematic representation of the synthesis of FeCo-MOF and schematic diagram of the photocatalytic degradation mechanism. Reproduced with permission of ref. [57], Copyright © 2023. Elsevier B.V. All rights reserved.

According to the hard–soft acid–base (HSAB) principle, 2 relatively intuitive strategies can be envisioned for constructing robust MOFs from the perspective of coordination bond strength: (a) hard acid/hard base combinations, such as high-valent metals (Zr^4+^, Cr^3+^, Al^3+^) with carboxylate (COO^−^) and (b) soft acid/soft base combinations, such as low-valent metals (Fe^2+^, Co^2+^, Ni^2+^, Zn^2+^) with pyrazolate (Pz) [52]. However, MOFs based on strong coordination bonds generally exhibit relatively poor self-healing capabilities, and the resulting MOF crystals are often unsuitable for single-crystal x-ray diffraction studies [53]. To date, only a few Pz-based MOFs utilizing 12-connected Ni_8_ clusters as SBUs have been reported. Li and colleagues [54] demonstrated a series of isostructural (2,12)-connected FCU (face-centered cubic) topology MOFs (JNU-212, JNU-213, JNU-214, and JNU-215) constructed from Ni_8_ clusters and 4 linear bipyrazole linkers (Fig. 7A). By systematically tuning the electron-accepting capacity of the pyrazole-bridging units from benzene to benzothiadiazole, benzoselenadiazole, and naphthoselenadiazole, the corresponding MOFs exhibited progressively enhanced photoelectrochemical performance. Notably, JNU-214 with benzoselenadiazole units displayed the most suitable Eg and highest ROS generation efficiency, establishing it as an exceptional photocatalyst for oxidative coupling of benzylamines (Fig. 7B). A plausible reaction mechanism for this photocatalytic aerobic benzylamine oxidation was proposed. Visible-light irradiation induces charge carrier generation and separation on JNU-214. The excited JNU-214* subsequently transfers energy to O_2_ via an ET mechanism, generating ^1^O_2_, which abstracts 2 H atoms from one benzylamine molecule to form a phenylmethanimine (PhCH=NH) intermediate and H_2_O_2_. Nucleophilic addition of another benzylamine molecule to PhCH=NH then yields the target product with concomitant release of NH_3_. Alternatively, photogenerated electrons reduce O_2_ to O_2_^•−^ through a SET mechanism, while photogenerated holes oxidize electron-rich benzylamine to amine radical cations (PhCH_2_NH_2_^•+^). Reaction between O_2_^•−^ and PhCH_2_NH_2_^•+^ generates PhCH=NH and H_2_O_2_, with subsequent nucleophilic addition of another benzylamine molecule to PhCH=NH, ultimately producing the product after NH_3_ release.

The systematic linker engineering demonstrated in these Ni₈ cluster-based MOFs represents a compelling and innovation-enabling design strategy for modulating charge transfer pathways and thereby tuning type I ROS generation efficiency, with JNU-214 exhibiting the highest photoactivity. However, the translational readiness of these materials remains uncertain because their stability and biocompatibility have not yet been evaluated. This gap is particularly important given that Ni^2+^ can pose toxicological risks (e.g., allergenicity and carcinogenicity), making metal leaching in biological environments a key concern. Although pyrazolate linkages are expected to form strong coordination bonds that could enhance resistance to hydrolysis, this assumption requires direct validation under physiological conditions (e.g., PBS and serum-containing media). In addition, the electron-accepting benzoselenadiazole and naphthoselenadiazole motifs introduce selenium-containing units, which may raise further safety concerns if degradation or leaching occurs, especially at elevated exposure levels.

The systematic linker engineering demonstrated in these Ni₈ cluster-based MOFs represents a compelling and innovation-enabling design strategy for modulating charge transfer pathways and thereby tuning type I ROS generation efficiency, with JNU-214 exhibiting the highest photoactivity. However, the translational readiness of these materials remains uncertain because their stability and biocompatibility have not yet been evaluated. This gap is particularly important given that Ni^2+^ can pose toxicological risks (e.g., allergenicity and carcinogenicity), making metal leaching in biological environments a key concern. Although pyrazolate linkages are expected to form strong coordination bonds that could enhance resistance to hydrolysis, this assumption requires direct validation under physiological conditions (e.g., PBS and serum-containing media). In addition, the electron-accepting benzoselenadiazole and naphthoselenadiazole motifs introduce selenium-containing units, which may raise further safety concerns if degradation or leaching occurs, especially at elevated exposure levels.

#### Bimetallic-MOF

Bimetallic-MOFs incorporate 2 distinct metal ions within their inorganic nodes, endowing them with synergistic effects and enhanced performance in catalysis, energy storage and conversion, and luminescent sensing [55]. For instance, Fe-MOFs exhibit poor degradation efficiency due to rapid recombination of electron–hole pairs under visible-light irradiation, while Co-MOFs suffer from low intrinsic activity owing to the inflexible electronic structure of Co [56]. In contrast, Fe–Co cluster-based MOFs, such as FeCo-MOF, synthesized by connecting Fe and Co with photon-harvesting organic ligands (2-aminoterephthalic acid), demonstrate improved optical properties [57]. The selectivity of FeCo-MOF is attributed to the presence of Fe-μ_3_-oxo clusters and their facilitation of direct excitation and ligand-to-metal charge transfer (LMCT) pathways. Meanwhile, the incorporation of Co ions enhances photocatalytic activity through abundant metal sites and a tunable porous structure. FeCo-MOF absorbs photon energy from sunlight equal to or exceeding its Eg energy. Following photon absorption, 3 types of excitations may occur: direct excitation via photon energy absorption by the metal clusters; LMCT, where electrons in the ligand are excited to the metal clusters; and the amino group oxidation and excitation to the metal clusters. Typically, electrons in the VB are photoexcited to the CB under solar irradiation, leaving holes in the VB. Due to the higher CB potential of FeCo-MOF, the excited electrons in the CB react with O_2_ to generate O_2_^•−^, requiring a significantly lower potential compared to •OH formation (Fig. 7C). The VB potential of FeCo-MOF is insufficient to produce •OH radicals, making O_2_^•−^ the sole ROS responsible for pollutant degradation. FeCo-MOF demonstrates superior photo-absorption capacity, reduced electron–hole recombination rates, high specific surface area, and optimal Eg energy and structure. Furthermore, ecotoxicity assessments reveal that degraded 2,4-D alters survival, development, reproduction, intracellular redox status, and stress response gene expression patterns in the *Caenorhabditis elegans* model.

FeCo-MOF exhibits higher photocatalytic activity than its monometallic analogs, most likely due to Fe/Co synergistic node chemistry that promotes charge separation and accelerates redox cycling. From a translational standpoint, however, bimetallic-MOFs are a double-edged sword: Fe and Co are essential trace elements, yet their uncontrolled release or dysregulated redox activity can trigger oxidative stress and toxicity. Notably, the intrinsic toxicity and biosafety of FeCo-MOF itself were not assessed, leaving critical questions unanswered.

### Covalent organic frameworks

COFs are crystalline porous polymers assembled from organic monomers linked by covalent bonds, yielding periodic structures with well-defined, ordered channels. Depending on the building blocks and linkage geometry, COFs can form either 2-dimensional (2D) layered frameworks (constructed via in-plane covalent connections and stacked through π–π interactions) or 3D networks that extend covalently in all spatial directions [58]. Their predictable topologies and highly tunable architectures have attracted broad interest across diverse applications [59]. In particular, the densely aligned columns and ordered π-stacking arrays in many COFs can facilitate directional charge migration, which is advantageous for photoredox processes [60]. Moreover, the modular diversity of building units—together with reversible bond formation during crystallization—enables precision design of crystalline order, pore environments, and electronic structures. COFs are especially attractive for photocatalytic and type I ROS-related applications because they integrate structural programmability with semiconductor-like photophysics. Robust covalent linkages impart high chemical and thermal stability, while extended π-conjugation within layers (and, in some systems, through-stack electronic coupling) supports efficient charge separation and charge carrier transport [61]. These features can translate into improved utilization of photogenerated electrons, which is a key determinant of O_2_ reduction and O_2_^•−^ formation in type I photochemistry [62]. Importantly, COFs offer a uniquely rational design space: By selecting and combining specific donor/acceptor motifs, tuning linkage chemistry, and controlling stacking order and pore microenvironments, researchers can systematically tailor topology, pore size, and functionality at the molecular level [63]. This “structure-to-function” programmability positions COFs as a mechanism-guided platform for optimizing O_2_^•−^ generation, rather than relying on empirical screening alone. Accordingly, this chapter focuses on how structural variations—including building units, linkage types, and framework architecture—govern the efficiency of O_2_^•−^ production.

#### Triazine-COF

**Fig. 8.**
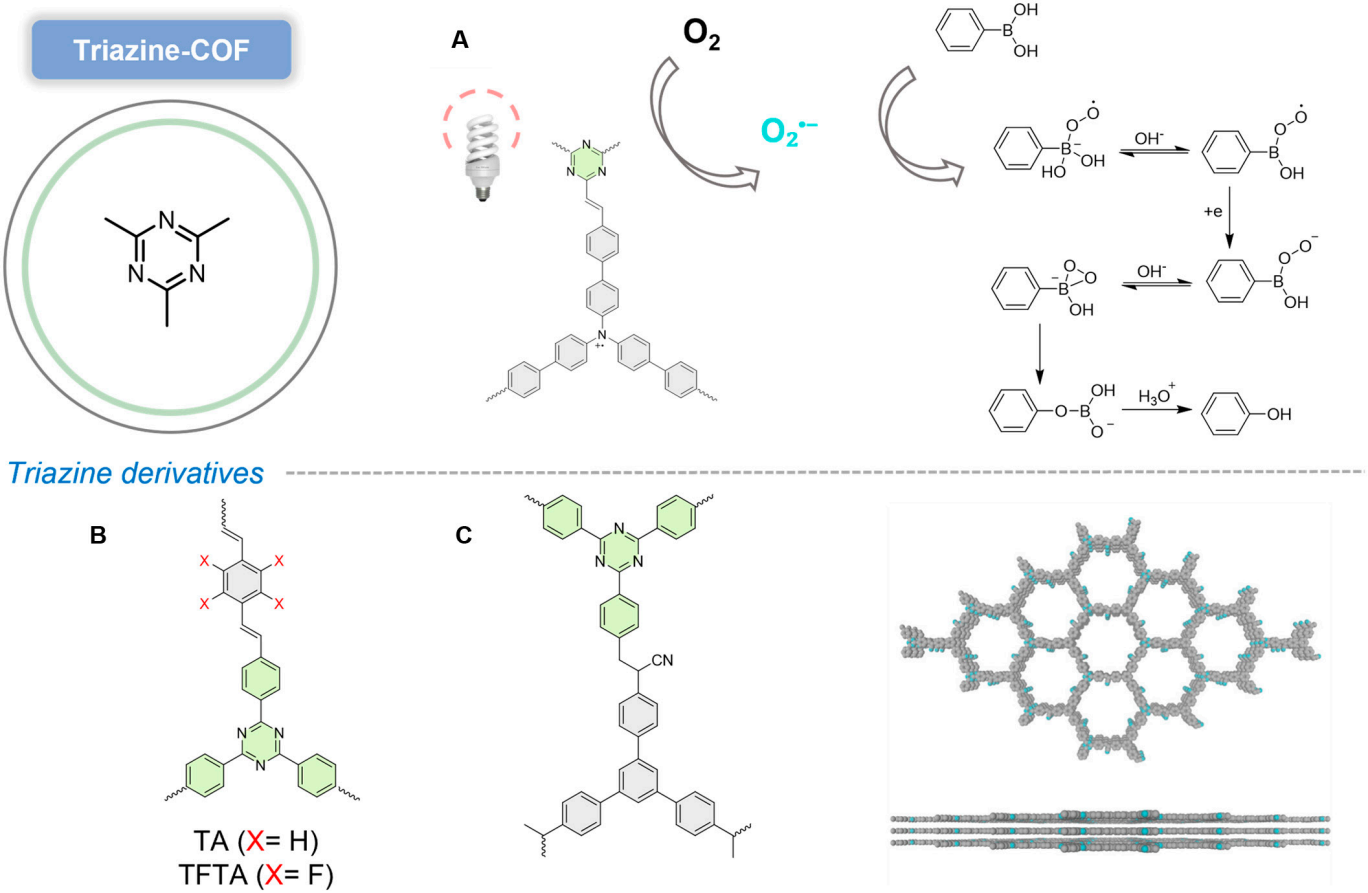
(A) Proposed catalytic mechanism of photocatalytic oxidative hydroxylation of aryl boronic acids without the assistance of a cocatalyst. Reproduced with permission of ref. [68], Copyright © 2022, Elsevier B.V. All rights reserved. (B) Synthesis design of H-COF, TF-COF, and TF50-COF. Reproduced with permission of ref. [69], Copyright © 2022, Wiley-VCH GmbH. (C) Synthesis design and AA stacking model of TA-sp^2^C-COF. Reproduced with permission of ref. [72], Copyright © 2023, Wiley-VCH GmbH.

COFs exhibit designable ordered porous structures with immense application potential in environmental and biomedical fields [64]. Notably, most COFs are constructed via reversible bonds such as imine or boroxine linkages. This reversibility allows mismatched bonds to undergo self-correction during synthesis, ultimately forming highly crystalline COFs under thermodynamic control [65]. Triazine-based COFs demonstrate exceptional promise in photocatalysis due to their high nitrogen content, visible-light responsiveness, and structurally tunable architectures [66]. However, these reversible bonds render COFs susceptible to hydrolysis and structural collapse in acidic/aqueous media, significantly limiting their practical applications [67]. Persistent radical-containing COFs show remarkable potential in photocatalysis, magnetism, and biomedicine. Two sp^2^ carbon-conjugated COFs (sp^2^C-COFs) incorporating stable triphenylamine radical cations (TPA^•+^) were synthesized [68], where efficient D–A heterojunctions enable radical simple generation through photoexcitation. Under visible light, electrons transfer from donor (TPA) to acceptor (triazine) units via PET, producing persistent TPA^•+^ and highly reactive electrons. The CB positions of these sp^2^C-COFs are sufficiently negative to reduce O_2_ into O_2_^•−^, while their visible-light absorption, tunability, and chemical stability make triazine-containing COFs particularly promising for photocatalysis (Fig. 8A).

Most reported covalent triazine frameworks (CTFs) suffer from low crystallinity or amorphous structures, which impair charge carrier generation/separation and limit photocatalytic activity. A fluorine-functionalized triazine-core imine-linked 2D COF (TF-COF) was designed to modulate electronic structures, extending visible-light absorption, enhancing charge separation, and promoting O_2_ adsorption [69]. With high crystallinity, mesopores, and abundant Lewis acid sites, this COF accelerates O_2_ chemisorption, facilitates charge transfer, and exhibits excellent photostability (Fig. 8B). Computational studies reveal that fluorine substitution strengthens π–electron interactions, improves crystallinity/porosity, and optimizes photogenerated charge transfer. Fully conjugated COFs, as an important subset of the COF family, are attracting increasing attention from researchers due to their exceptional chemical stability and superior electron delocalization properties [70]. However, their synthesis remains challenging owing to the low reversibility of vinylene linkages [71]. By modulating alkali dosage and temperature, a novel triazine-based sp^2^ carbon-conjugated COF (TA-sp^2^C-COF) was successfully constructed via Cs_2_CO_3_-mediated synthesis [72]. Furthermore, the influence of modulation factors on the chemical and optoelectronic properties of TA-sp^2^C-COF was thoroughly investigated. TA-sp^2^C-COF adopts an eclipsed AA stacking structure with uniform micropores (Fig. 8C). Its blue-light-driven photocatalytic activity for selective oxidative coupling of amines with oxygen was established, with O_2_^•−^ identified as the dominant species in imine formation. Upon blue-light excitation, TA-sp^2^C-COF generates separated electrons and holes. The photogenerated electrons are captured by O_2_ during the reaction, sequentially forming O_2_^•−^. Meanwhile, the photogenerated holes oxidize benzylamine to benzylamine radical cations, which are further oxidized by O_2_^•−^ to produce intermediate benzaldimine. Subsequent nucleophilic attack by another benzylamine molecule on benzaldimine yields n-benzylbenzaldimine products.

#### Porphyrin-COF

**Fig. 9.**
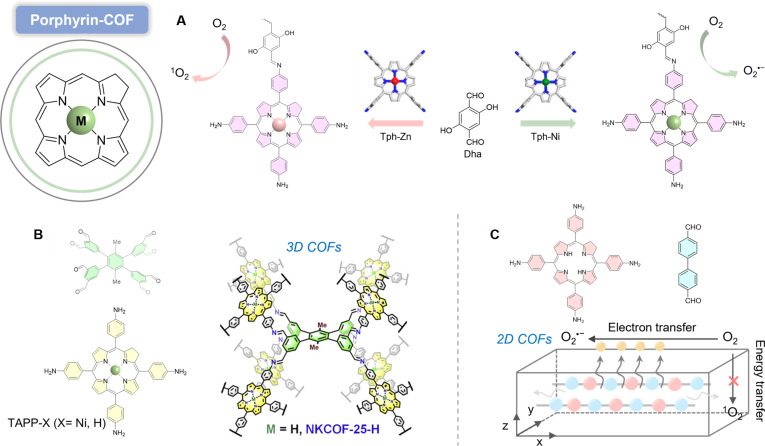
(A) Synthesis and crystal structure of DhaTph-M (M=Ni and Zn) and diagram representation of their discriminative O_2_ activation selectivity to O_2_^•−^ under visible-light irradiation. Reproduced with permission of ref. [76], Copyright © 2020, American Chemical Society. (B) Band structure diagram for NKCOF-25-X. Reproduced with permission of ref. [79], Copyright © 2022, Elsevier Inc. (C) Illustration of synthesis of 367 and schematic illustration of active surface exposure ratios and the O_2_ activation pathway in COF-367-2D. Reproduced with permission of ref. [81], Copyright © 2024, American Chemical Society.

Porphyrins and their derivatives are unique chromophores with intriguing photophysical properties, offering significant potential for photocatalysis [73]. The layered π–π stacking structure of porphyrin-based COFs facilitates interlayer charge carrier transport, endowing them with exceptional performance [74]. Porphyrinic framework materials demonstrate outstanding photocatalytic capabilities due to their abundant metal-active sites, tunable crystal structures, and high specific surface areas [75]. For example, porphyrin-based COFs (DhaTph-M, M=Zn, Ni) have proven to be ideal models for modulating excitonic effects, depending on the absence or presence of different metals at the porphyrin center [76]. Compared with DhaTph-Zn, the incorporation of Ni^2+^ in COFs promotes exciton dissociation into hot carriers under photoexcitation (Fig. 9A). Consequently, DhaTph-Ni exhibits superior photocatalytic activity in O_2_^•−^-mediated boronic acid hydroxylation reactions, where Ni^2+^ at porphyrin centers accelerates exciton dissociation and charge transfer processes to generate O_2_^•−^ from O_2_. This O_2_^•−^ generation is recognized as an electron transfer process: When DhaTph-Ni is photoexcited, excitons dissociate around Ni centers due to energetic disorder, followed by electron transfer to O_2_ molecules adsorbed on Ni sites, producing O_2_^•−^. A 0.07 e^−^ depletion at Ni centers to the Zn center clearly demonstrates that the Ni^2+^ in the porphyrin center is prone to promote electron transfer to O_2_ for the production of O_2_^•−^.

3D COF materials, due to their interpenetrating and interconnected pore structures, abundant active sites, and high structural stability, have shown potential applications in various fields such as gas separation and heterogeneous catalysis [77]. However, due to challenges such as the limited types of building blocks and difficulties in structural elucidation, only a few 3D COF topologies have been reported so far. The development of new building blocks and topologies is urgently needed [78]. Based on this, Zhang and colleagues [79] utilized an octaldehyde-based monomer with D2h symmetry and synthesized 2 porphyrin-based 3D COFs (NKCOF-25-H, NKCOF-25-Ni) through an [8+4] connectivity (Fig. 9B). The authors combined continuous rotation electron diffraction (cRED) technology with theoretical simulations to confirm that the COFs exhibit an SCU (simple cubic) topology. Due to their excellent light absorption capabilities and well-matched band structures, the NKCOFs demonstrated enhanced photocatalytic capabilities for generating O_2_^•−^.

Dimensional modulation of COFs profoundly impacts absorption, charge separation, and surface reactivity [80]. Zhou and colleagues [81] proposed a strategy for selectively producing ROS by adjusting the dimension of porphyrin-based COF (COF-367). Reversible protonation of imine bonds enables structural transformation from 3D frameworks to 2D sheets (Fig. 9C). The 2D COFs expose more active sites, enhancing O_2_ interaction and electron transfer to favor O_2_^•−^ generation. The reduced charge migration resistance in ultrathin COFs enables rapid photoelectron migration to active surfaces, accelerating O_2_^•−^ production rates. Simplified internal structures, fewer defects, and shorter charge migration paths in 2D COFs further optimize photocatalytic efficiency. The photogenerated carriers in existing porphyrin-based COFs are prone to rapid recombination, resulting in low ROS generation efficiency [82]. By simultaneously protonating and metalizing porphyrin units, exciton migration can be effectively suppressed, and the separation of photogenerated charge carriers can be promoted, thereby overcoming these limitations.

#### Pyrene-COF

**Fig. 10.**
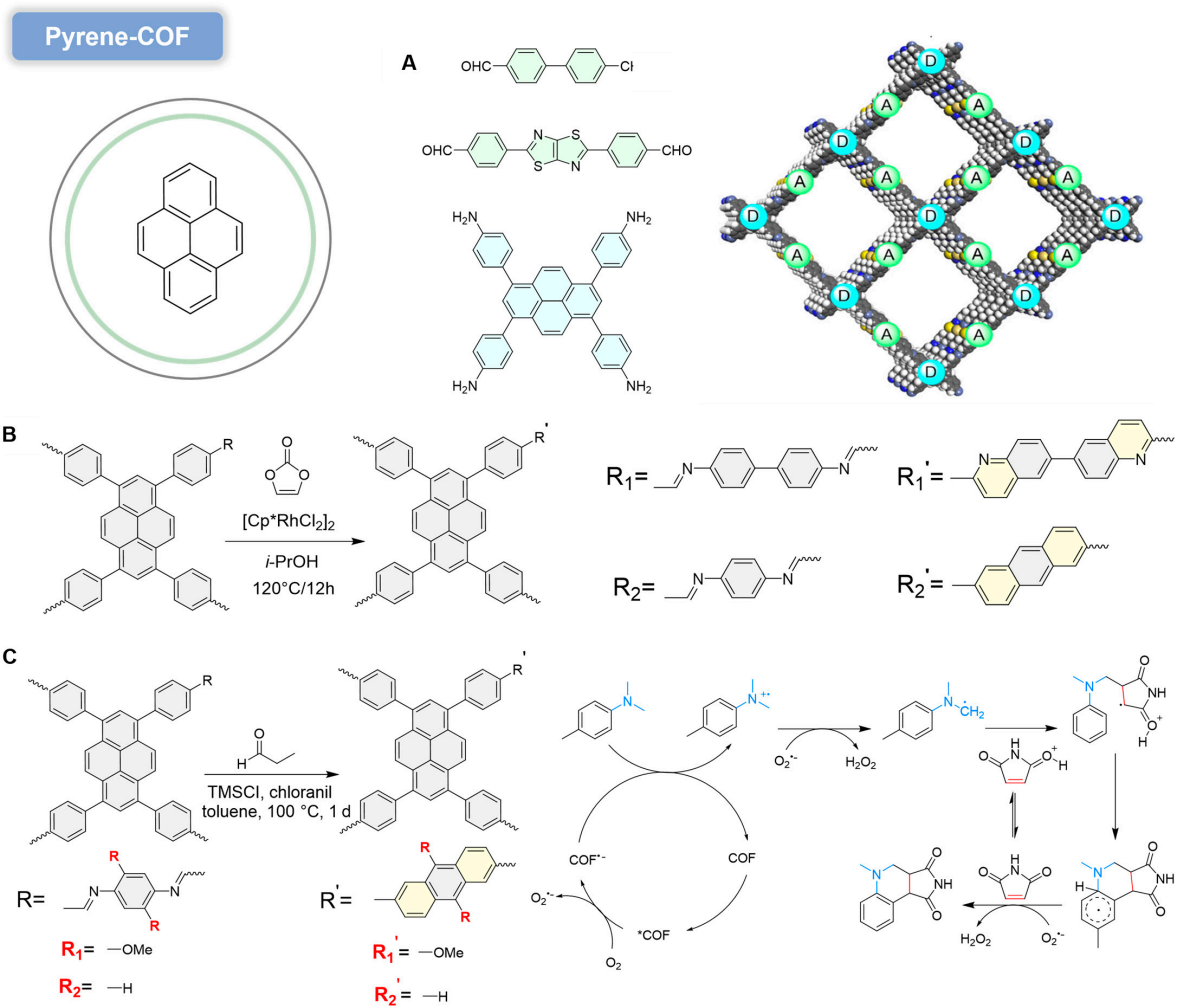
(A) Synthesis of PyTz-COF and structure of the D–A COF. Reproduced with permission of ref. [86], Copyright © 2020, Wiley-VCH GmbH. (B) Construction of nonsubstituted quinoline-linked NQ-COF (TfppyPh) and NQ-COF (TfppyBp) via Rh-catalyzed annulation. Reproduced with permission of ref. [88], Copyright © 2022, Wiley-VCH GmbH. (C) Synthetic schematic of QLCOF-n through TMSCl catalysis and a proposed mechanism for photocatalytic annulation. Reproduced with permission of ref. [90], Copyright © 2025, American Chemical Society.

Pyrene, a widely studied aromatic hydrocarbon, exhibits unique optical and electronic properties [83]. Pyrene-based molecules possess strong absorption capabilities and long excited-state lifetimes, endowing pyrene-based COFs with potential applications in photocatalysis and optoelectronic devices [84]. By precisely controlling COF structures, efficient charge transport can be achieved, preventing charge accumulation and recombination [85]. For example, a novel D–A structured 2D COF (PyTz-COF) [86], constructed from electron-rich pyrene and electron-deficient thiazolo[5,4-d] thiazole, demonstrates exceptional photocatalytic activity in O_2_^•−^-mediated oxidative coupling of arylmethyl amines (Fig. 10A). Under visible-light irradiation, PyTz-COF absorbs photons to excite electrons from the VB to the CB, generating electron–hole pairs. The photoelectrons migrate to the COF surface, reducing O_2_ to O_2_^•−^, which oxidizes primary amines to imines. Simultaneously, photogenerated holes are consumed by water molecules or sacrificial agents, suppressing charge recombination. The resulting imine intermediates desorb from the COF surface, enabling continuous catalytic turnover. The dynamic nature of imine linkages (C=N) in COFs often compromises their stability [87]. To address this, a rhodium-catalyzed [4+2] cycloaddition strategy was developed to irreversibly convert C=N bonds into nonsubstituted quinoline linkages, yielding chemically stable nonsubstituted quinoline-linked COFs (NQ-COFs) [88]. This approach achieves high C=N conversion rates without oxidants while preserving crystallinity, topology, and porosity (Fig. 10B).

Post-synthetically modified NQ-COFs exhibit enhanced visible-light absorption and charge carrier separation efficiency due to improved in-plane π-conjugation. The extended conjugation enables efficient light-to-energy conversion, promoting electron excitation to the CB. The spatially separated electrons and holes migrate to active sites, where electrons reduce O_2_ via a SET pathway to generate O_2_^•−^, while holes oxidize substrates to yield target products. Photocatalytic metal-free α-amino C–H cyclization is a pioneering strategy for obtaining heterocyclic compounds. Imine-linked COFs (ILCOFs), known for their photon excitation potential, remain underutilized in C–H cyclization due to inherent photochemical instability and inefficient C–H activation [89].

Cao and colleagues [90] proposed a novel locking strategy, utilizing chlorotrimethylsilane-catalyzed cyclization to transform ILCOF-n (imine-linked COFs) into stable QLCOF-n (quinoline-locked COFs) with methylquinoline motifs, thereby establishing it as a robust photocatalytic platform for metal-free α-amino C−H cyclization. This structural modulation preserves crystallinity while enhancing structural robustness and photoelectric properties compared to parent ILCOFs. Superior catalytic efficiency and stability of QLCOF-n over ILCOF-n were confirmed through scale-up/recycling experiments and substrate derivatization with 22 samples. Given that exploration of cyclization parameters revealed O_2_’s promotional effect on catalysis, a possible reaction mechanism was proposed: After photoexcitation, the generation of excited COF^•+^ from COF* correlates with O_2_^•−^ formation from O_2_, potentially mutually enhanced. This step is crucial as it leads to a key amino carbon radical via C–H bond activation. Specifically, COF^•+^ converts to COF under light induction, prompting 2’s transition to excited state 2^•+^, followed by dehydrogenation to generate the amino carbon radical. This radical undergoes radical addition with protonated 1a. The resulting intermediate then experiences intramolecular cyclization, subsequently undergoing aerobic dehydrogenation to yield final product 3 (Fig. 10C). Additionally, O_2_^•−^ reacts with released H^+^ to generate H_2_O_2_, thereby enhancing the turnover efficiency of photocatalytic cyclization. Mechanistic insights highlight the critical roles of amino carbon and O_2_^•−^ radicals in cyclization. This work establishes quinoline-locked QLCOFs as durable photocatalysts for sustainable C–H functionalization.

#### 1,3,5-Triaoyl-triphenylphenol-COF

**Fig. 11.**
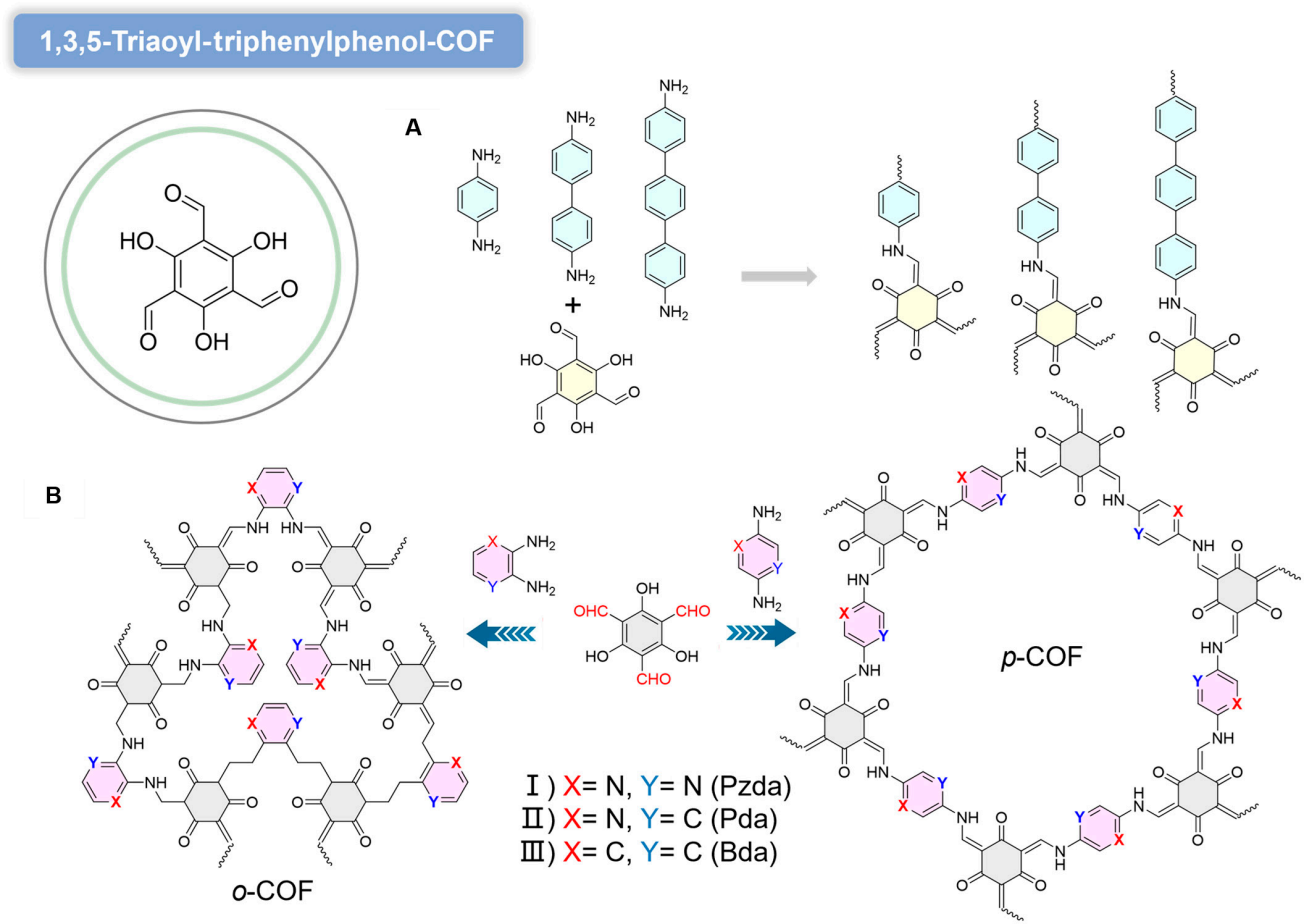
(A) Design of the 3 β-ketoenamine COFs with different linker lengths. Reproduced with permission of ref. [91], Copyright © 2022, Elsevier B.V. All rights reserved. (B) Calculated band structures of the 3 β-ketoenamine COFs. Reproduced with permission of ref. [92], Copyright © 2024, The Authors. Angewandte Chemie International Edition published by Wiley-VCH GmbH.

1,3,5-Triformylphloroglucinol contains 3 aldehyde crosslinking. They proposed that this microneedle could serve groups in its molecular structure, enabling condensation reactions with amine monomers to form β-ketoenamine linkages for COF construction [91]. The abundance of aldehyde sites allows 2,4,6-triformylphloroglucinol (TFP) to react with diverse amines, creating COFs with tunable structures and properties. β-Ketoenamine-linked COFs (TpPa-COF, TpBD-COF, and TpDT-COF) exhibit excellent crystallinity and π–π stacking interactions, providing efficient pathways for photogenerated charge separation and transfer (Fig. 11A) [92]. The strong conjugation of β-ketoenamine bonds not only ensures structural robustness but also endows COFs with adjustable porosity for enhanced reactant/product accommodation. Increasing the length of connecting arms extends the π-conjugation system in COFs, leading to widened optical Egs and improved visible-light absorption. Consequently, COFs with longer linkers generate more photogenerated electron–hole pairs. Typically, COFs can be constructed through reactions between C2-symmetric building blocks bearing para- reactive groups and C3, C4, and C6-symmetric functionalized building blocks. Derived COFs linked via para covalent bonds (*p*-COFs) generally exhibit high symmetry. For example, a *p*-COF formed from phenyl-para-diamine and 1,3,5-triformylphloroglucinol is used for H_2_O_2_ photosynthesis. By doping N and S=O groups into the *p*-COF to promote the separation of photoinduced electron–hole pairs, the modified *p*-COF demonstrates enhanced photocatalytic H_2_O_2_ production efficiency. Notably, by replacing the para-diamine with a low-symmetry ortho-diamine, COFs with ortho covalent bonds (*o*-COFs) or metal-doped COFs can also be formed. For bare *p*- and *o*-COFs with identical composition, their molecular and electronic structures may differ. However, the influence of the linkage position in *o*- and *p*-COFs on their photocatalytic performance, particularly in H_2_O_2_ photocatalysis, has rarely been reported. Studies by Lang and colleagues demonstrate that in designing COF photocatalysts with long-lasting high activity without the need for sacrificial agents, linker positions are of great importance. Ortho-linked COFs (*o*-COFs) demonstrate superior activity and stability compared to para-linked counterparts (*p*-COFs) despite identical compositions [93]. The uniform distribution of active sites in *o*-COFs enhances catalyst–reactant interactions, while their β-ketoenamine bonds serve as active centers for 4e^−^ oxidation reactions (Fig. 11B).

#### Others

**Fig. 12.**
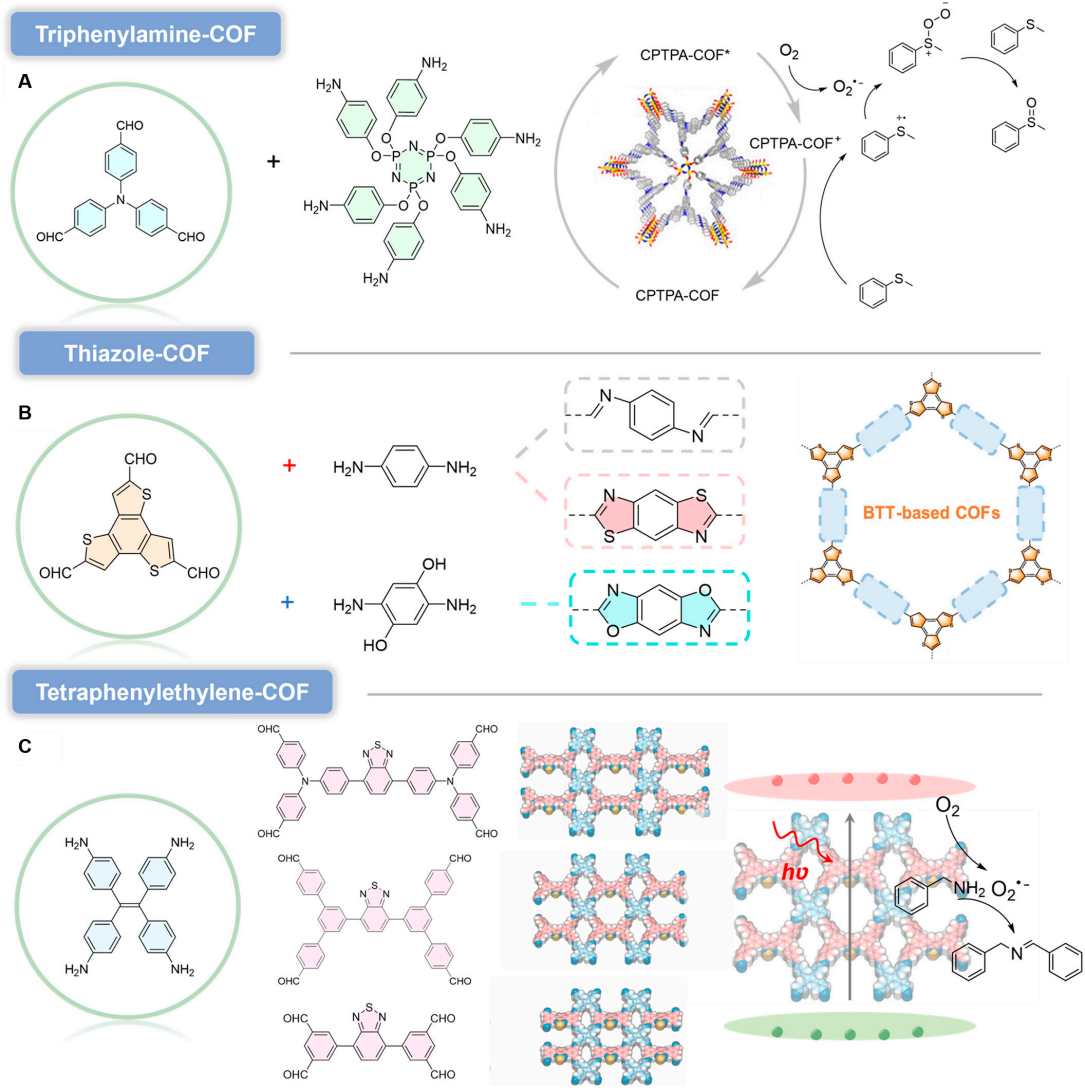
(A) Preparation scheme of CPTPA-COF and mechanism diagram of photocatalytic degradation toward paracetamol by CPTPA-COF. Reproduced with permission of ref. [95], Copyright © 2022, American Chemical Society. (B) Preparation scheme of COF-A, COF-O, and COF-S. Reproduced with permission of ref. [96], Copyright © 2024, The Author(s). (C) Design, synthesis, and crystal structures of JUC-675, JUC-676, and JUC-677, and proposed reaction mechanisms for JUC-676. Reproduced with permission of ref. [101], Copyright © 2024, Wiley-VCH GmbH.

Triphenylamine is an important photochromic molecule. Due to its high thermal stability and fatigue resistance, it has been widely employed as a photoactive unit in the fabrication of various photosensitive materials for applications in chemical sensing, bioimaging, and PDT [94]. A photoactive COF was designed and constructed by incorporating photoactive triphenylamine groups into the framework (CPTPA-COF) [95]. This approach not only successfully formed a highly crystalline and stable framework but also revealed the photoactivity of TPA-based building blocks (Fig. 12A). Electron paramagnetic resonance measurements demonstrated that the COF acts as an efficient photocatalyst capable of generating O_2_^•−^. Under irradiation, CPTPA-COF effectively triggers the generation and separation of electron–hole pairs. The photoexcited electrons rapidly transfer to the surface or channels of CPTPA-COF, enabling the rapid reduction of O_2_ to O_2_^•−^. Subsequent studies confirmed the high efficiency, selectivity, and reusability of CPTPA-COF in the photocatalytic aerobic oxidation of sulfides. In the reaction system, sulfides are oxidized by separated CPTPA-COF^+^ holes to form sulfur-centered radicals. These radicals capture O_2_^•−^ to generate dipolar persulfoxide intermediates, which then react with another sulfide molecule to ultimately yield sulfoxide products.

Thiazole, a 5-membered heterocyclic structure with significant ring strain, exhibits rigid structural characteristics [86]. This rigidity enhances the stability of COFs, preventing deformation or cleavage during reactions and ensuring structural integrity and catalytic activity. Tong and colleagues [96] successfully transformed conventional ILCOFs into oxazole/thiazole-linked COFs (COF-O and COF-S) through a simple one-pot synthesis strategy by converting reversible imine bonds into rigid oxazole/thiazole bonds (Fig. 12B). Furthermore, based on ultrafast spectroscopy experiments and theoretical calculations, oxazole/thiazole bond-linked COFs effectively regulate the exciton dissociation behavior on COFs by modulating the π-conjugation of the framework and local charge polarization. Compared with reversible imine bonds, the rigid oxazole/thiazole bonds in COFs also enhance the robustness and structural stability of the COF skeletons. The thiazole bond in COF-S with an optimal C^2^p state efficiently enhances the activity of the *o*-phthalaldehyde units and modulates the O_2_ adsorption energy barrier, thereby exhibiting superior degradation performance toward paracetamol under visible-light irradiation. Compared to reversible imine linkages, thiazole-linked COFs (COF-S) display superior chemical stability, maintaining structural integrity even after exposure to acidic or alkaline solutions [97]. The optimal π-conjugation and localized charge separation properties of thiazole-linked COFs (COF-S) facilitate efficient charge separation and transfer in excited states. Enhanced charge separation efficiency improves the photocatalytic activity of COFs, enabling the generation of more ROS (e.g., O_2_^•−^) and thereby increasing O_2_ activation rates. The tetraphenylethylene unit possesses electron-donating properties that enhance the electron-donating capacity of COF frameworks [98]. This promotes the separation efficiency and mobility of photogenerated charge carriers, leading to improved photocatalytic activity [99]. Furthermore, the introduction of tetraphenylethylene units modulates the band structure of COFs, resulting in a narrower band gap. This modification not only broadens the absorption range of COFs but also enhances photocatalytic efficiency [100]. Additionally, D–A structured COFs typically exhibit broader visible-light absorption. Liu et al. [101] successfully synthesized a series of D–A COFs (JUC-675, JUC-676, JUC-677) with excellent crystallinity. These materials not only possess high specific surface area, porosity, outstanding thermal stability, and chemical stability but also exhibit unique electronic and optical properties. The incorporation of triphenylamine units enhances the electron-donating ability of the framework, improving the separation efficiency and migration rate of photogenerated charge carriers, thereby effectively boosting photocatalytic activity. When applied as catalysts in a photocatalytic tandem system for H_2_O_2_ production and BA (Brønsted acids) oxidation coupling, JUC-675 demonstrates the most outstanding photocatalytic performance compared to currently reported COF-based photocatalysts. The D–A COFs possess reduced band gaps, enabling more efficient solar light absorption and photoelectron generation. These photoelectrons can transfer to sulfur atomic sites, promoting O_2_^•−^ formation (Fig. 12C). Notably, sulfur atomic sites exhibit the strongest O_2_ adsorption energy and the lowest activation energy for O_2_^•−^ generation, indicating their superior activity in producing O_2_^•−^.

COFs provide exceptional structural and electronic tunability, and recent reports have already demonstrated encouraging in vivo PDT efficacy, underscoring their translational promise. However, COF performance in biological settings is highly chemistry-dependent, with stability and biocompatibility governed by both linkage motifs and building-block composition. In particular, widely used imine linkages, although synthetically convenient, can undergo hydrolysis under acidic and/or aqueous conditions, potentially undermining framework integrity in biological fluids. A key innovation emerging in the field is post-synthetic or linkage-conversion engineering to “lock” COFs into more hydrolytically robust architectures—such as quinoline-linked (e.g., NQ-COFs and QLCOFs) or thiazole-linked (e.g., COF-S) frameworks—which show markedly improved resistance to degradation; notably, QLCOFs retaining crystallinity after 72 h in PBS highlights a meaningful advance toward physiological robustness. Biocompatibility evidence is also advancing but remains uneven: TPS-PEG (a COF nanoparticle) has been evaluated with hemocompatibility assays and in vivo histology, indicating minimal systemic toxicity, while Ptp-Fe (Fe^3+^-incorporated porphyrin centers) shows enhanced phototoxicity with acceptable dark toxicity in cancer cell lines. For most COF platforms, however, essential translational data are still missing, including colloidal stability in serum, protein corona formation and its impact on uptake, identity/toxicity of degradation products, and long-term in vivo fate. Establishing standardized, quantitative biocompatibility and biointerface protocols—paired with rational linkage stabilization strategies—would enable meaningful cross-study comparisons, sharpen structure–property–biosafety design rules, and accelerate the identification of clinically actionable COF PSs for PDT.

### Hydrogen-bonded organic frameworks

HOFs have emerged as an important complement to conventional porous organic frameworks (POFs) by enabling framework construction through weak, reversible intermolecular interactions rather than permanent covalent or coordination bonds [102]. This bonding mode imparts excellent self-assembly and error correction during crystallization, allowing HOFs to be synthesized under mild conditions while still achieving high crystallinity and structural robustness [103]. As a result, HOFs have attracted increasing interest in applications ranging from photocatalysis to proton conduction [104]. Notably, many HOFs exhibit sufficient chemical and thermal stability to maintain structural integrity and functional performance even under relatively harsh operating conditions, supporting their practical use [105]. A distinctive advantage of HOFs is their highly tunable structure–property space: Subtle changes in synthesis conditions (e.g., solvent system, concentration, temperature, or additives) can modulate framework topology, pore environment, morphology, and interfacial properties, thereby enabling application-specific optimization. From a photoredox perspective, their ordered crystalline packing and well-defined surface microenvironments can promote charge separation and suppress electron–hole recombination, ultimately improving photocatalytic efficiency [106]. Moreover, the ability of HOFs to retain chemical stability over repeated photocatalytic cycles is particularly valuable for real-world implementation, where durability and recyclability are essential performance metrics.

#### Pyrene-HOF

A major challenge associated with HOFs lies in their weak hydrogen bonding, which limits the structural stability of HOFs [107]. This instability compromises the advantages of HOFs and hinders their widespread application in photocatalytic hydrogen evolution [108]. To enhance the stability of HOFs, researchers have adopted various strategies, including the formation of multiple hydrogen bonds and the utilization of π–π interactions [109]. Nevertheless, weak charge transfer capability remains a challenge for this class of organic semiconductor photocatalysts. Zhou et al. [110] designed a HOF with hydrophilic 1D microporous channels, 1,3,6,8-tetrakis (*p*-carboxyphenyl) pyrene (H_4_TBAPy), as a model system for photocatalytic hydrogen evolution, demonstrating that the strategy of shortening the distance between exciton coupling to the catalyst surface can effectively enhance the utilization efficiency of photogenerated excitons. The results revealed that under illumination, photogenerated excitons rapidly transfer to the inner surfaces of adjacent micropores, forming a transfer path as short as 1.88 nm, thereby significantly improving exciton utilization efficiency. Inspired by the self-assembly approach of the supramolecular organic catalyst Py-HOF, Li and colleagues [111] designed a novel pyrene-based S-scheme HOF/COF heterojunction (H_4_TBDPy) using a rapid solution method. Owing to strong hydrogen bonds and π–π interactions, H_4_TBDPy molecules readily aggregate to form a suitable band structure, thereby establishing a supramolecular heterojunction with Py-COF.

**Fig. 13.**
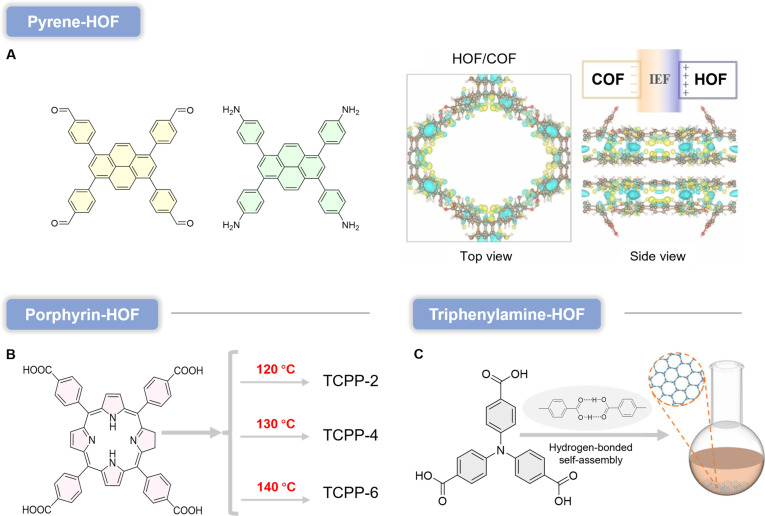
(A) Top view and side view of the charge density difference (CDD) of the HOF/COF. Reproduced with permission of ref. [119], Copyright © 2024, Published by Elsevier B.V. on behalf of Chinese Chemical Society and Institute of Materia Medica, Chinese Academy of Medical Sciences. (B) Preparation conditions for the HOFs (TCPP-2, TCPP-4, TCPP-6). Reproduced with permission of ref. [124], Copyright © 2019, American Chemical Society. (C) Schematic diagram of HOF-16 for the photocatalytic oxidation of silane. Reproduced with permission of ref. [127], Copyright © 2024, American Chemical Society.

The appropriate interfacial matching between the dual pyrene-based frameworks, enabled by their similar chemical structures and sufficient interfacial contact, promotes efficient interfacial charge transfer. This facilitates the effective separation of photogenerated electrons and holes. These photogenerated electrons may be captured by certain defects or active sites in the catalyst, forming O_2_^•−^. Pyrene-based materials possess extensive conjugated π-systems, enabling efficient visible-light absorption and generation of photogenerated electrons and holes. These materials can interact with other molecules via hydrogen bonds to form HOFs [112]. This self-assembly process occurs rapidly, and the structure and morphology of HOFs can be controlled by simply altering the solvent, facilitating their application in photocatalysis [113]. However, the quantum yield of HOF is still limited by the transfer of photogenerated charges to the surface [114,115]. As previously mentioned, under irradiation, H_4_TBAPy facilitates the rapid transfer of photo-generated excitons to the inner surface micropores of adjacent structures. This process shortens the transmission paths, thereby significantly enhancing exciton utilization. The excitonic effect and strong interlayer confinement in self-assembled frameworks enhance ROS yield. Under photoexcitation, HOF structures generate excited-state electrons and holes, which migrate to the framework surface [116]. These charge carriers react with adsorbed O₂, activating them to produce ROS that drive substrate oxidation [117]. The ordered and porous nature of HOFs promotes the adsorption of O₂ and substrates, enhances charge separation, and accelerates ROS generation [118]. For example, a pyrene-based HOF (PFC-1), constructed from H₄TBAPy, exhibits significantly improved ROS production efficiency due to excitonic effects and interlayer confinement, making it highly promising for photocatalytic oxidation (Fig. 13A) [119]. Under visible light, PFC-1 transitions from its ground state ([PFC-1]) to an excited state ([PFC-1]*), transferring energy to O_2_ to generate ROS. These ROS oxidize substrates to yield target products. The integration of D–A architectures into hydrogen-bonded frameworks (D–A HOFs) further enhances PDT efficiency. With methanol slowly diffusing into the *N, N*-dimethylacetamide solution of H_4_TBAPy, an isoreticular HOF (PFC-111) was obtained [120]. Unlike conventional covalent linkages, PFC-111, a D–A HOF, synthesized using hydrogen-bonded D–A pairs, features a reduced energy gap (ΔE_st_) between singlet and triplet excited states. This promotes ISC and boosts ROS generation. Upon photon absorption, the lowest excited singlet state of PFC-111 is populated. The small ΔE_st_ facilitates efficient transition to the lowest triplet state, whose energy is transferred to O_2_ via ET, generating ROS. The high exciton binding energy of PFC-111 suppresses charge transfer pathways, directing more energy toward ROS production. This structural design highlights the potential of HOFs in advanced photocatalytic and therapeutic applications.

#### Porphyrin-HOF

Porphyrins, with their 18π-electron conjugated systems and strong charge transfer capabilities, enable efficient visible-light-driven photocatalytic reactions [121]. By utilizing diverse hydrogen-bonding groups, various porous porphyrin-based HOFs can be constructed, providing abundant active sites to enhance the separation and transfer of photogenerated charges [122]. Additionally, the stable structure of porphyrins and their resistance to chemical corrosion allow them to adapt to diverse reaction environments. Due to their exceptional optoelectronic properties, porphyrins are selected as building blocks for creating porous HOFs with permanent porosity and robust chemical stability [123]. The higher nitrogen content in porphyrin-based HOFs improves photoelectron transport, facilitates reduction reactions, and enhances electrical conductivity, thereby promoting the separation and migration of photogenerated charges. By adjusting reaction temperatures, the binding positions and quantities of capping molecules with porphyrin derivatives can be controlled, modulating the framework rigidity and porosity of HOFs, which directly impacts their photocatalytic performance. Luo and colleagues [124] reported the controlled synthesis of meso-tetrakis (carboxyphenyl) porphyrin (TCPP)-based HOFs via a “counterstrategy” of blocking the strong hydrogen-bonded building units on the TCPP backbones. Under irradiation, TCPP molecules are excited to the singlet state. The S_1_ state reacts with O_2_ to generate ROS, which subsequently oxidize substrates (e.g., 9,10-diphenylanthracene) into corresponding products (Fig. 13B). The quantity and binding positions of encapsulated molecules influence the molecular stacking of TCPP and the pore structure of the HOF, thereby affecting the separation efficiency of photogenerated electron–hole pairs, substrate adsorption, and reactant diffusion—all of which collectively determine the photocatalytic performance.

#### Triphenylamine-HOF

The triphenylamine molecule, with its highly 3-dimensional structure, efficiently absorbs and transfers photo-energy to generate reactive species such as ROS [125]. HOFs constructed from TPA units exhibit compact interlayer stacking and confinement effects, which enhance the generation efficiency of ROS, thereby boosting photocatalytic activity [126]. The incorporation of TPA units also improves the fluorescence intensity and quantum yield of HOF materials, enhancing their light-harvesting and utilization capabilities for photocatalytic processes. For instance, the TPA-based HOF (HOF-16) demonstrates remarkable advantages in the photocatalytic hydroxylation of silanes [127]. Compared to its monomeric counterpart, HOF-16 achieves tighter interlayer alignment and confinement effects, significantly increasing ^1^O_2_ production efficiency (Fig. 13C). As a key reactive species in photocatalytic oxidation, the high-yield generation of O_2_ is essential for improving reaction efficiency. Under 360- to 365-nm UV irradiation, HOF-16 transitions from its ground state to an excited state. The excited state [HOF-16]* is quenched by O_2_, generating ^1^O_2_. This ^1^O_2_ then undergoes a hydrogen atom transfer (HAT) reaction with silane substrates, producing a silicon radical I and a hydroxyl radical ([HOO]*). Radical I reacts with [HOO]* to form intermediate II, which hydrolyzes to yield the final silanol product. Notably, HOF-16 exhibits exceptional photocatalytic performance under mild conditions, characterized by high reaction efficiency, low catalyst loading, and excellent reusability. This highlights its potential for sustainable and efficient photocatalytic applications.

In biological fluids, the hydrogen-bond networks that stabilize HOFs can be disrupted by water, competitive hydrogen-bond donors/acceptors (e.g., serum proteins), and fluctuations in pH and ionic strength—factors that may accelerate framework disassembly, aggregation, or cargo/ligand release. To date, most HOF studies have been confined to organic solvents or pure water, and systematic stability data in PBS, cell culture media, or serum are largely unavailable. Although PFC-1 and PFC-111 have been examined in aqueous solutions, their behavior in complex biological media remains unverified. Equally importantly, biocompatibility evidence is almost entirely absent. No cytotoxicity assays, hemocompatibility testing, or in vivo evaluations have been reported for the HOFs discussed here, despite the fact that common HOF chromophore building blocks (e.g., porphyrins, pyrenes, and triphenylamines) can exhibit intrinsic bioactivity and may pose toxicity risks upon dissolution or metabolic processing. This gap highlights a major opportunity—and a clear innovation direction—for the field: Moving HOFs from “chemically elegant” materials to translation-ready PDT platforms supported by quantitative biointerface and biosafety data. Before HOFs can be credibly advanced for PDT, several foundational studies are urgently needed: (a) quantitative disassembly and stability kinetics in PBS, serum-containing media, and relevant pH/ionic-strength windows; (b) identification, quantification, and toxicological profiling of dissociation products; (c) standardized in vitro cytotoxicity, hemocompatibility, and cellular uptake assessments (including dark-state oxidative stress); and (d) stability-by-design strategies to preserve crystallinity and function in vivo, such as strengthening auxiliary interactions (π–π stacking, hydrophobic packing, ion pairing, or metal coordination) and developing hybrid architectures (e.g., HOF/COF or core–shell constructs) that combine HOF processability with COF-like robustness.

### Supramolecular organic frameworks

SOFs are multidimensional networked assemblies constructed through noncovalent (supramolecular) interactions rather than permanent covalent or coordination bonds [128]. They typically form via the self-assembly of organic building blocks driven by hydrogen bonding, van der Waals forces, π–π stacking, hydrophobic effects, and, in some systems, auxiliary coordination interactions [129]. This “soft-bonded” construction mode offers 2 distinctive advantages: (a) structural adaptivity (error correction during assembly) and (b) modular tunability, enabling rapid optimization of framework packing, pore environments, and interfacial properties. Many SOFs exhibit porous or channel-containing architectures that expose abundant accessible sites and facilitate reactant diffusion, thereby improving photocatalytic turnover. Importantly, by rationally selecting and combining organic chromophoric units, the optical absorption of SOFs can be programmed to better overlap with the solar spectrum, enhancing light-harvesting efficiency and overall photon utilization [130]. In addition, the ordered supramolecular packing can promote exciton dissociation and accelerate photogenerated electron–hole separation, reducing recombination losses and ultimately boosting catalytic performance [131]. Collectively, these features position SOFs as a design-flexible photoredox platform, where functionality can be tuned through building-block engineering and controlled supramolecular organization rather than relying solely on compositional changes.

#### Pyrene-SOF

**Fig. 14.**
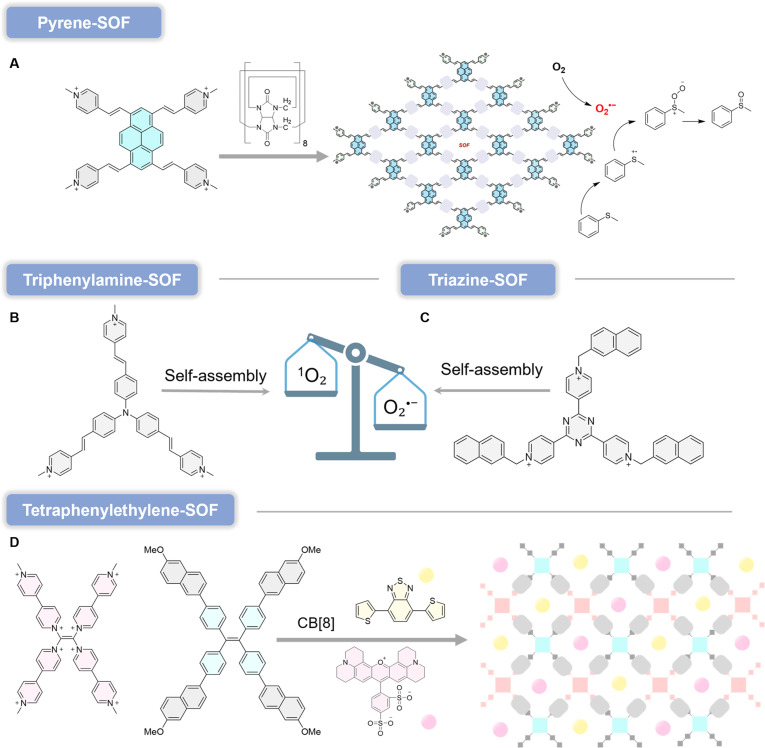
(A) Illustration of the construction of the SOF between Pmvp and CB[8]. Reproduced with permission of ref. [134], Copyright © 2024, Royal Society of Chemistry. Illustrates the schematic representation of TP-SOF (B) and TN-SOF (C). Reproduced with permission of ref. [136], Copyright © 2023, Elsevier Inc. All rights reserved. (D) Illustration of the artificial LHS based on MV-TPE, NA-TPE, and CB[8]. Reproduced with permission of ref. [141], Copyright © 2024, Royal Society of Chemistry.

Pyrene derivatives offer multiple, readily addressable substitution sites and a rigid, extended π-conjugated core. This combination enables precise molecular-level structural programming and, upon assembly, provides robust pathways for electronic delocalization, making pyrene motifs particularly attractive building blocks for constructing SOFs [132]. Leveraging this high designability, SOF properties can be rationally tuned by controlling substituent identity and position as well as intermolecular packing, thereby introducing programmable functions [133]. A pyrene-based SOF (Pmvp) constructed in aqueous solution via host–guest interactions between a pyrene derivative and CB[8] exhibits periodic porous structures, homogeneous solubility, and the ability to regulate ROS generation [134]. Compared to Pmvp, the confined architecture of SOF reduces the conformational freedom of pyrene derivatives and lowers their excited-state energy, thereby suppressing ^1^O_2_ production while enhancing O_2_^•−^ generation (Fig. 14A). Upon irradiation, SOF absorbs photon energy, exciting electrons from the ground state to form electron–hole pairs. The excited electrons transfer to ligands or solvent molecules, such as O_2_, generating O_2_^•−^ via electron transfer. These radicals subsequently react with substrates to drive oxidation processes. For instance, in thiol oxidation reactions, O_2_^•−^ oxidizes thiols to sulfoxides or sulfones. This mechanism enables SOF to demonstrate exceptional photocatalytic activity in aqueous systems, achieving 87% conversion in the aerobic oxidation of thioethers and 99% conversion in the oxidative hydroxylation of arylboronic acids.

#### Triphenylamine-SOF

The conjugated system in triphenylamine structures enables visible-light absorption, particularly in the blue and green regions, making triphenylamine derivatives suitable for visible-light-driven aqueous photocatalytic reactions [135]. For instance, methylated vinylpyridine-functionalized triphenylamine derivatives efficiently generate ^1^O_2_, enabling photocatalytic oxidation of thioanisole in aqueous solutions [136]. Compared to the monomeric TP-3Py, the confinement effect of cucurbit[8]uril (CB[8]) significantly enhances O_2_^•−^ generation efficiency, showcasing promising applications in photocatalytic oxidation. A novel 2D SOF (TP-SOF) constructed via host–guest interactions between a TP-3Py and CB[8] demonstrates efficient O_2_^•−^ production under green-light irradiation (Fig. 14B). Upon irradiation, TP-SOF transitions from the ground state to an excited state ([TP-SOF]*). In the presence of diisopropylethylamine (DIPEA), [TP-SOF]* undergoes electron transfer to form [TP-SOF]^•−^ and DIPEA^•+^. The generated [TP-SOF]^•−^ is oxidized by O_2_ to produce O_2_^•−^, which reacts with arylboronic acid to form intermediate I. Subsequent HAT yields intermediate II and an iminium cation. Hydrolysis of the iminium cation produces acetaldehyde and diisopropylamine, while intermediate II undergoes rearrangement to release hydroxide ions, forming intermediate III. Final hydrolysis of III releases boric acid and yields the target product. The cavity of CB[8] encapsulates the methylated vinylpyridine units of TP-3Py, restricting their mobility. This confinement effect promotes intramolecular electron transfer, enhancing O_2_^•−^ generation efficiency. Meanwhile, interactions between CB[8] and the electron-deficient pyridine moieties of TP-3Py strengthen charge transfer processes and facilitate excited-state electron transfer from TP-3Py to O_2_, further boosting O_2_^•−^ production. Additionally, the TP-SOF architecture effectively disperses active sites, preventing aggregation and improving catalytic activity.

#### Triazine-SOF

Modification of the pyridine groups in the triazine derivative through the introduction of substituents or functional groups enables tailored properties and functionalities, expanding its potential for constructing SOFs with diverse applications [137]. It exhibits good water solubility, allowing its assembly and use in aqueous environments. Additionally, triazine derivatives inherently possess photocatalytic activity, generating both ^1^O_2_ and O_2_^•−^, which lays the foundation for designing SOFs with photocatalytic capabilities [138]. Upon forming an SOF (TN-SOF) via interactions with CB[8], the confinement effect imposed by the macrocyclic host and framework structure significantly enhances ^1^O_2_ and O_2_^•−^ generation efficiency (Fig. 14C) [139]. The cavity of CB[8] encapsulates the methylnaphthalene units of TN-3Ny, restricting molecular motion and thereby reducing nonproductive quenching of ^1^O_2_ and O_2_^•−^ radicals. This prolongs their lifetimes, enabling more effective participation in photocatalytic oxidation reactions and improving overall efficiency. As a PS, TN-SOF absorbs photo-energy under irradiation, transitioning to an excited state ([TN-SOF]*). Subsequent electron transfer with DIPEA generates [TN-SOF]^•−^ and DIPEA^•+^. The [TN-SOF]^•−^ species reacts with O_2_ to produce O_2_^•−^. The 2D layered structure of TN-SOF provides abundant active sites for O_2_^•−^ generation and transport, further enhancing radical production. Importantly, TN-SOF demonstrates superior photocatalytic activity compared to the TN-3Ny monomer, attributed to its exceptional ability to generate ^1^O_2_ and O_2_^•−^. This structural and functional synergy highlights the potential of SOF-based systems in advanced photocatalytic applications.

#### Tetraphenylethylene-SOF

The hydrophobicity of tetraphenylethylene molecules makes them poorly soluble in aqueous solutions. This hydrophobic nature facilitates the formation of stable SOF structures through interactions with other hydrophobic units, strengthening binding forces between tetraphenylethylene units and other components [140]. The emission spectrum of TPE (tetraphenylethylene) can overlap with the absorption spectra of other units, enabling ET processes [141]. TPE units act as energy donors, transferring energy to other components or dye molecules, a mechanism exploitable for building artificial light-harvesting systems (LHSs) to enhance photo-utilization efficiency (Fig. 14D). For example, a guest interaction between a TP-3Py and CB[8] demonstrates that 3D SOF was constructed via host–guest interactions between tetraphenylethylene derivatives (MV-TPE and NA-TPE) and CB[8]. This SOF exhibits strong fluorescence emission in water and serves as an energy donor for an artificial LHS [142]. By introducing energy-accepting dyes dibenzothiophene (DBT) and sulforhodamine 101 (SR101), a 2-step ET cascade—from SOF to DBT, then to SR101—was achieved, resulting in white-light emission. The SOF’s ordered porous structure facilitates light harvesting and promotes interactions between substrates and catalysts. The fluorescent SOF efficiently captures photo-energy and transfers it to DBT and SR101. These dyes further relay energy to O_2_, enhancing O_2_^•−^ generation. Concurrently, the SOF acts as an electron donor, transferring electrons to DBT and SR101, which then serve as electron acceptors to mediate electron transfer to O_2_, further boosting O_2_^•−^ production. Notably, the SOF + DBT + SR101 system demonstrates significantly higher O_2_^•−^ generation efficiency compared to individual components (SOF, DBT, or SR101 alone), underscoring the synergistic effects of this integrated design.

SOFs assembled through host–guest recognition offer a distinctive platform for aqueous photocatalysis, combining dynamic, self-adaptive assembly, water compatibility, and confinement/organization effects that can promote charge transfer and enhance O_2_^•−^ generation. These features represent a clear innovation opportunity for PDT, particularly in settings where conventional PSs suffer from poor solubility or aggregation. Nevertheless, the stability and biosafety of SOFs in biologically relevant environments remain largely unvalidated, which currently limits their translational potential. A central concern is that the noncovalent interactions underpinning SOFs may be perturbed by dilution, competitive binding (proteins, metabolites, endogenous guests), and physiological ionic strength. For example, although TP-SOF and TN-SOF show improved ROS generation in water, their performance in serum-containing media—where proteins may compete for CB[8] binding and/or disrupt π–π stacking—has not been examined. Biocompatibility data are also scarce: Neither the framework components (e.g., vinylpyridine-functionalized triphenylamines and methylnaphthalene derivatives) nor the CB[8] macrocycle has been systematically evaluated in these SOF formulations for cytotoxicity, uptake, or in vivo behavior. Given their intrinsic suitability for aqueous operation, SOFs could be especially attractive for topical or localized PDT (e.g., skin infections or superficial lesions), but this application space requires a robust safety and stability evidence base. Accordingly, critical next steps include (a) quantifying assembly integrity and disassembly kinetics in PBS and cell culture media [e.g., via DLS (dynamic light scattering), FRET (fluorescence resonance energy transfer), and/or spectroscopy under dilution and salt challenges]; (b) cytotoxicity and dark-ROS screening in relevant cell lines; (c) evaluating protein corona formation and its consequences for colloidal stability, cellular interactions, and ROS generation; and (d) developing stability-by-design strategies that preserve the advantages of supramolecular assembly while improving in vivo robustness—most notably post-assembly “locking” approaches (e.g., covalent capture/crosslinking) to transform dynamic SOFs into physiologically durable, translation-ready type I ROS-generating systems.

#### Halogen-bonded organic frameworks

**Fig. 15.**
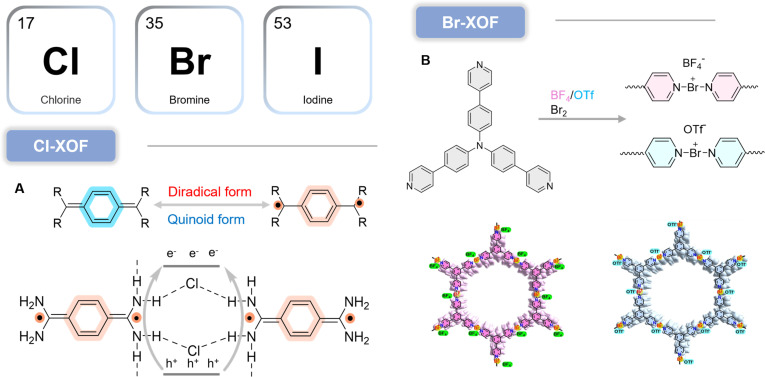
(A) Resonance structures of the quinoid and diradical forms and schematic diagram of the mechanism for XHOF-TAQ. Reproduced with permission of ref. [146], Copyright © 2022, The Author(s). (B) Construction of BrPy_2_BF_4_/OTf and [N···Br···N]^+^-bridged XOF(Br) TPy_2_BF_4_/OTf. Reproduced with permission of ref. [147], Copyright © 2024, Wiley-VCH GmbH.

Halogen bonding (XB) is a directional noncovalent interaction that arises when an electron-deficient region on a halogen atom approaches an electron-rich site (typically a Lewis base) [143]. In classical XB, a halogen atom bound to an electron-withdrawing substituent develops a σ-hole—an electropositive region opposite the covalent bond—which enables attractive donors, and halonium ions (X^+^) possess a positively charged, *p*-orbital-derived electrophilic site that can simultaneously engage 2 Lewis bases, forming a highly directional, near-linear 3-center-4-electron linkage, often denoted as [D···X^+^···D] [144,145]. This motif is particularly attractive for supramolecular construction because it can act as a node-like connector that is strong, geometrically predictable, and synthetically programmable. Against this backdrop, XOFs have emerged as a distinctive branch of POFs, expanding the framework “bond toolbox” beyond covalent and coordination chemistry. Their hallmark is the use of halogen-bond connectivity to create adaptable porous architectures, which can unlock functions that are difficult to access in conventional frameworks. For example, using chloride ions as connecting nodes to assemble 7,7,8,8-tetraaminoquinodimethane (TAQ) affords a 3D crystalline material with high stability, tunability, and notable optical properties [146]. In these XHOF materials (X = halogen), halide ions serve as nodes within [Cl^−^···H] hydrogen-bonded motifs (Fig. 15A). The synergistic coupling of halide ions with electron-rich organic ligands strengthens the noncovalent network, endowing the framework with robust solvent resistance, thermal stability, and environmental durability. Moreover, the photoinduced singlet open-shell diradical character of TAQ can supply highly reactive excited electrons, imparting XHOF-TAQ with strong photoredox capability and high effective redox potential.

More broadly, organic frameworks often face an inherent trade-off between bond strength (stability) and bond dynamics (processability and crystallization), motivating the development of innovative linkages that deliver stability without sacrificing mild, efficient synthesis. In this context, halonium-based XOFs are especially compelling. However, constructing bromine(I)-bridged frameworks is challenging because Br(I) species are typically highly moisture-sensitive. Bai et al. [147] addressed this by constructing a stable 2D bromine(I)-bridged halogen-bonded framework, XOF(Br)·BrPy_2_·BF_4_/OTf, based on the sensitive yet highly directional [N···Br···N]^+^ linkage. Strikingly, once embedded into the extended framework, XOF(Br) exhibits excellent chemical and thermal stability: It retains its 2D architecture in various organic solvents and remains stable in water across a broad pH range, whereas the molecular precursor BrPy_2_·BF_4_/OTf rapidly decomposes even in trace water (Fig. 15B). This “stability-by-architecture” effect substantially improves aqueous compatibility and expands the practical scope of bromine(I) halogen-bond chemistry. Functionally, XOF(Br)·BrPy_2_·BF_4_/OTf can serve as an efficient mild oxidant for benzyl alcohol oxidation in aqueous media. Notably, the counterion plays a nontrivial role: Compared with BF_4_^−^, OTf^−^ can provide improved stability and reactivity, underscoring counterion engineering as an additional design lever in XOF chemistry. Collectively, these advances demonstrate that XOFs do more than diversify porous frameworks structurally—they introduce an unconventional, supramolecularly programmable reactivity platform, enabling green and efficient transformations under mild conditions, a capability that is rarely achieved with other framework classes.

XOFs are an emerging and largely underexplored family of porous materials with distinctive photophysical and redox characteristics. A particularly compelling advance is the “stability-by-architecture” concept demonstrated by XOF(Br): Despite the inherent moisture sensitivity of the molecular [N–Br^+^–N] halogen(I) linkage, the framework preserves its 2D structure in water over a broad pH window. This unexpected aqueous resilience is highly significant because it elevates halogen-bonded assemblies from chemically fragile molecular motifs to potentially bio-relevant solid-state platforms, enabling application scenarios that would be unrealistic for discrete halogen(I) complexes. At the same time, biocompatibility and biosafety remain completely uncharted for XOFs, and several high-priority risks must be addressed before biomedical translation can be considered. First, any release of halogen(I) species (Br^+^/I^+^) could introduce acute reactivity and toxicity concerns. Second, XOF integrity in complex biological media is unknown; nucleophiles abundant in vivo (e.g., thiols and amines) may attack the halogen bond and accelerate degradation. Third, the intrinsic cytotoxicity of the organic components (e.g., TAQ and pyridine-derived linkers) must be evaluated, particularly if partial disassembly leads to molecular exposure. Finally, the degradability, biodistribution, and clearance of these frameworks are entirely unexplored. To date, no in vitro or in vivo biosafety data have been reported for any XOF, making toxicity assessment the most urgent bottleneck. Importantly, this gap also defines a clear innovation opportunity. If XOFs can be rendered physiologically stable and biocompatible through rational design (e.g., architectural stabilization, protective coatings, or controlled-release mitigation), their unusual photoredox behavior—such as the photoinduced diradical character of TAQ—could enable nonclassical, type I PDT pathways that are difficult to access with conventional PSs. In this sense, XOFs currently sit at the earliest discovery stage: The materials concept is highly promising, but biosafety validation and mechanistic toxicology are the decisive, yet still entirely unanswered, questions on the route to biomedical application.

### General design principles for type I organic frameworks

The diverse case studies surveyed above show that, despite the wide structural diversity of organic frameworks, the rational design of high-performance type I PSs consistently converges on 2 core and highly general engineering axes: (a) bandgap/band-edge engineering [148] and (b) charge-transport engineering [149]. The first axis dictates light-harvesting capability and the thermodynamic feasibility of electron transfer to O_2_, while the second controls how efficiently photogenerated carriers are separated, transported, and delivered to reactive interfaces. Bandgap and band-edge engineering is typically implemented through D–A motif construction, metal-node selection, and linker functionalization, with the explicit goal of positioning the CB minimum (or LUMO-derived level) sufficiently negative relative to the O_2_/O_2_^•−^ redox couple, thereby enabling productive electron transfer and radical initiation [150]. Charge-transport engineering, by contrast, focuses on maximizing carrier utilization by suppressing recombination and accelerating interfacial kinetics. Common approaches include extending π-conjugation, enforcing ordered stacking, tuning excitonic effects, constructing heterojunctions, and optimizing linkage chemistry to promote long-lived charge-separated states and fast electron delivery to adsorbed O_2_ (or other acceptors). Notably, the most effective frameworks do not optimize these axes in isolation; rather, they co-engineer thermodynamics and kinetics. Representative strategies include pairing strongly electron-accepting linkers with robust metal clusters that promote charge separation, or integrating dual type I/type II motifs within a single architecture to preserve phototoxicity across heterogeneous oxygenation [151]. This “2-axis” framework therefore highlights a key innovation theme of organic frameworks: Their architectural programmability enables simultaneous control over energy-level alignment and carrier migration, allowing ROS output to be designed—not merely observed. By distilling these recurring motifs into transferable principles, our analysis provides a mechanism-centered design roadmap for next-generation type I organic framework PSs, helping move the field from case-by-case demonstrations toward predictive, structure-guided innovation (Table 1).

**Table 1.** Summary of key parameters for type I organic framework PSs

Material	Type	Bandgap (eV)	Superoxide (O_2_^•−^) generation	Self-healing efficiency	Ref.
JNU-204	Zn-MOF	2.35	Confirmed (EPR)	≥3 cycles	[39]
Zn_2_(BODIPY)_2_(TDC)_2_	Zn-MOF	2.2	Confirmed (EPR)	≥6 cycles	[40]
LCU-600	Al-MOF	3.25	Confirmed (EPR)	≥5 cycles	[43]
UiO-68-BTDB	Zr-MOF	2.55	Confirmed (EPR)	≥5 cycles	[48]
JNU-214	Ni-MOF	1.71	Confirmed (EPR)	≥3 cycles	[54]
FeCo-MOF	Bimetallic-MOF	1.73	Confirmed (EPR)	≥5 cycles	[57]
TF-COF	Triazine-COF	2.09	Confirmed (EPR, NBT): 1.2 × 10^−5^ M	≥5 cycles	[69]
TA-sp^2^C-COF	Triazine-COF	2.5	Confirmed (EPR, quenching)	≥4 cycles	[72]
DhaTph-Ni	Porphyrin-COF	2.46	Confirmed (EPR, NBT): 8.23 × 10^−6^ M	≥5 cycles	[76]
NKCOF-25-Ni	Porphyrin-COF	1.78	Confirmed (EPR, NBT): 96% NBT degradation in 70 min	≥10 cycles	[79]
PyTz-COF	Pyrene-COF	2.20	Confirmed (by photocatalytic amine coupling)	≥5 cycles	[86]
TpDT-COF	β-Ketoenamine-COF	2.25	Confirmed (EPR, quenching)	≥3 cycles	[92]
CPTPA-COF	Triphenylamine-COF	2.41	Confirmed (EPR)	≥5 cycles	[95]
COF-S	Thiazole-COF	2.19	Confirmed (ESR)	≥5 cycles	[96]
JUC-675	D–A COF	2.08	Confirmed (EPR, quenching)	≥8 cycles	[101]
PFC-1	Pyrene-HOF	~	~	~	[119]
PFC-111	D–A HOF	1.8	~	~	[120]
TCPP-6	Porphyrin-HOF	~	~	~	[124]
HOF-16	Triphenylamine-HOF	~	~	≥5 cycles	[127]
Pmvp-SOF	Pyrene-SOF	~	Confirmed (EPR, TMPD)	~	[163]
TP-SOF	Triphenylamine-SOF	~	Confirmed (EPR, TMPD)	~	[166]
TN-SOF	Triazine-SOF	~	Confirmed (EPR, TMPD)	~	[169]

## Type I Organic Framework in PDT

Ongoing efforts to combine diverse metal ions with modular organic ligands have continuously expanded the library of organic framework materials. Among them, the biocompatibility of MOFs provides a key prerequisite for biomedical translation. More broadly, the rigid, periodic, and ordered architectures of organic frameworks offer clear photophysical advantages: They can improve light utilization and suppress aggregation-caused quenching (ACQ), a major limitation of many molecular PSs [152]. Importantly, framework platforms that intrinsically favor type I ROS generation (and can be further optimized by structural or surface engineering) have already shown strong potential in anticancer and antibacterial applications [153]. These advances underscore an innovation trend in the field: Organic frameworks are evolving from passive PS carriers into mechanism-engineerable photoredox platforms capable of programming ROS pathways for challenging biological microenvironments.

### Antitumor

**Fig. 16.**
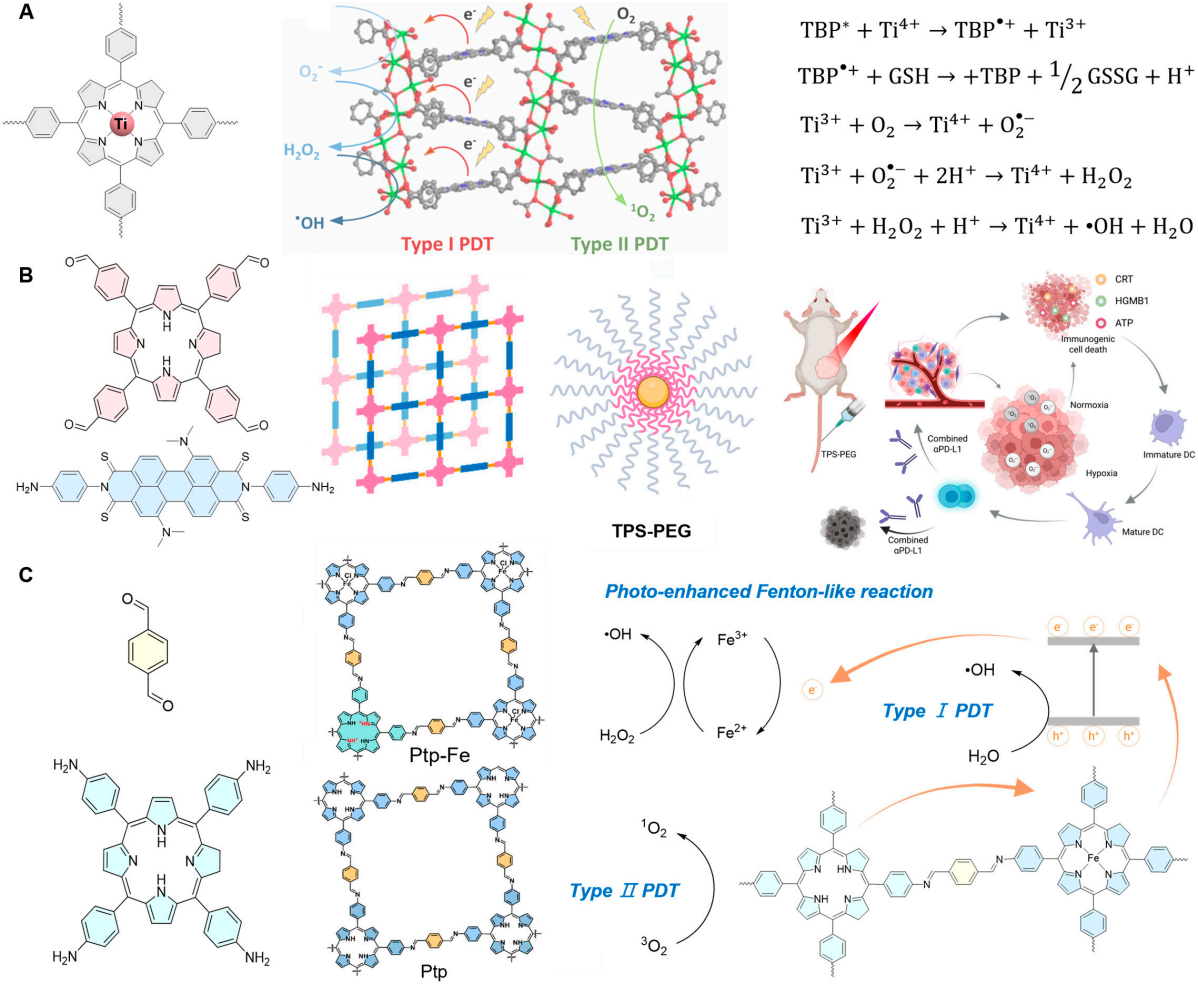
(A) Schematic showing both type I and type II PDT enabled by Ti-TBP. Reproduced with permission of ref. [158], Copyright © 2019, American Chemical Society. (B) Schematic illustration for the preparation of COF nano-PSs with staggered type I and II PS motifs and synergistic type I and II PDT to overcome heterogeneous distribution of normoxia and hypoxia in tumors and to induce efficient ICD for photodynamic immunotherapy. Reproduced with permission of ref. [159], Copyright © 2024, American Chemical Society. (C) Proposed mechanisms for ^1^O_2_ and •OH generation by Ptp-Fe under light irradiation. Reproduced with permission of ref. [160], Copyright © 2024, American Chemical Society. Figure created with Biorender.com.

Hypoxia is a hallmark of solid tumors, arising from rapid tumor proliferation and inadequate O_2_ delivery due to abnormal vasculature [2,154]. This hypoxic microenvironment markedly compromises conventional O_2_-dependent type II PDT. Hypoxia is a hallmark of solid tumors, arising from rapid tumor proliferation and inadequate O_2_ delivery due to abnormal vasculature. This hypoxic microenvironment markedly compromises conventional O_2_-dependent type II PDT, which primarily relies on sensitizing O_2_ to generate cytotoxic ^1^O_2_. As a result, type II PDT often eliminates tumor cells mainly in normoxic regions, with limited efficacy in hypoxic areas—contributing to residual disease, recurrence, and metastasis. To address this limitation, a research team reported a nanoscale MOF (nMOF), Ti-TBP (5,10,15,20-tetra(p-benzoato)porphyrin), that enables hypoxia-tolerant type I PDT by producing multiple ROS (^1^O_2_, O_2_^•−^, H_2_O_2_, and •OH) under irradiation (Fig. 16A) [155]. Confocal imaging and flow cytometry (apoptosis assays) confirmed extensive apoptosis after Ti-TBP treatment, and MTS (CellTiter 96 AQueous One Solution Cell Proliferation Assay)/live-dead analyses yielded IC_50_ values of 3.4 ± 0.7 μM and 7.8 ± 2.4 μM, respectively. Mechanistically, photoexcited TBP* transfers electrons to Ti^4+^ nodes, generating Ti^3+^ and TBP^•+^ species. Ti^3+^ subsequently reduces O_2_ to O_2_^•−^, which can disproportionate to H_2_O_2_; H_2_O_2_ is then converted into highly cytotoxic •OH via Fenton-type reactions. Innovation-wise, this nMOF exemplifies a “charge-relay + ROS-cascade” design, converting limited O_2_ availability into amplified radical chemistry—an approach that is inherently better aligned with hypoxic tumors.

Because O_2_ distribution is also spatially heterogeneous within solid tumors, materials that maintain ROS output across varying O_2_ levels are particularly valuable. COFs offer high stability, adjustable porosity/size, and controllable ordered stacking, supporting their use in drug delivery and phototherapy [156]. Notably, the rigid and periodic arrangement of PS motifs—together with interlayer offset stacking—can isolate chromophores, suppress ACQ, and improve photostability, thereby strengthening PDT performance [157]. Using perylenetetracarboxylic dianhydride (PTCDA) derivatives, a type I PS organic linker (PDA-S) was designed via intramolecular D–A coupling and sulfur substitution to enhance ISC and electron transfer capability [158]. Through Schiff-base condensation between amino groups in PDA-S and aldehyde groups in a type II porphyrin PS [tetraldehyde phenyl porphyrin (TPP)], followed by PEGylation, COF NPs (TPS-PEG) were obtained [159]. TPS-PEG integrates both type I and type II photochemistry: Under irradiation, it generates ^1^O_2_ and O_2_^•−^ to eradicate cancer cells in normoxic regions, while in hypoxic regions it continues to produce O_2_^•−^ through type I pathways, sustaining cytotoxicity. Notably, robust ROS production can induce immunogenic cell death (ICD), alleviating tumor immunosuppression; when combined with immune checkpoint blockade (ICB), TPS-PEG achieves enhanced systemic antitumor immunity (Fig. 16B). The innovation here is a single, ordered COF platform that couples hypoxia-tolerant ROS chemistry with immunotherapy synergy, enabling both local ablation and suppression of distant tumors.

Porphyrins and derivatives remain the most widely used clinical PSs [2], and porphyrin-based COFs are promising nano-PS candidates. However, many porphyrin COFs suffer from rapid charge recombination, limiting ROS yields. To overcome this, a dual protonation–metallation strategy was developed. For example, simultaneous protonation and Fe^3+^ metallation of porphyrin units in a 2D imine-linked porphyrin COF (Ptp-Fe) markedly improved PDT efficacy [160]. Protonation induces a hyperporphyrin-like state, redshifting and strengthening Q-band absorption to improve tissue penetration and light utilization. Meanwhile, Fe^3+^-porphyrin centers disrupt exciton migration, suppress excited-state quenching, and prolong excited-state lifetimes, thereby enhancing ROS generation. The IC_50_ values obtained from MTS/viability analysis in CT26 and 4T1 cell lines were 9.2 ± 0.7 μ M and 17.8 ± 2.4 μ M, respectively. Electron transfer from photoexcited protonated porphyrins to Fe^3+^ activates type I pathways and photo-enhanced Fenton + catalytic amplification strategy that directly targets the main bottleneck of porphyrin COFs (Fig. 16C).

In parallel, Wang and colleagues [161] designed a ternary D–A–D metal-containing COF (CuTD-COF). Owing to its narrower bandgap and stronger photoreactive radical effect, CuTD-COF exhibits longer excited-state lifetimes and enhanced light trapping relative to a binary D–A analog (CuT-COF), enabling effective tumor suppression. Mechanistic and DFT (density functional theory) analyses indicate that the D–A–D architecture introduces multiple electron transfer pathways that enhance intralayer charge transport, reduce exciton binding energy, accelerate carrier separation/migration, and suppress recombination—collectively increasing ROS output. This work highlights a key innovation direction for COF phototherapy: engineering exciton behavior and band dispersion as a rational route to predictable ROS control. Photogenerated carrier separation and migration work together to suppress charge recombination, thereby enhancing ROS generation. CuTD-COF exhibits potent photodynamic cytotoxicity in vitro and demonstrates outstanding antitumor efficacy in HCT116 xenograft models. Through integrated experimental and DFT findings, researchers identified distinct exciton behaviors governing ROS generation in the 2 D–A–D MCOFs (metal-containing COF), attributable to their band energy dispersion and O_2_ adsorption energy. This work not only diversifies the optoelectronic structures of MCOFs but also advances the development of optical cancer therapy.

Recent advances in MOF- and COF-based photomedicine highlight a clear developmental trajectory: from O_2_-dependent type II PDT toward hypoxia-tolerant type I systems, and further toward multifunctional platforms integrating phototherapy with chemotherapy or immunotherapy. One emerging strategy focuses on mechanistic reprogramming of PSs. For example, the introduction of electron transfer mediators such as thymoquinone enables porphyrin-based MOFs to shift from ET-dominated type II PDT to electron transfer-driven type I pathways [162]. This transformation fundamentally changes the dominant ROS from ^1^O_2_ to O_2_^•−^, thereby mitigating hypoxia-induced efficacy loss. Such mediator-driven modulation offers a simple yet broadly applicable approach for engineering hypoxia-tolerant PSs without redesigning the core framework. In parallel, structural innovation at the framework level has enabled deeper tissue penetration and improved therapeutic integration. Multiphoton-active COFs with D–π–A architectures demonstrate strong nonlinear optical responses, allowing long-wavelength excitation and deep-tissue imaging while simultaneously supporting PDT [163]. When combined with hypoxia-activated prodrugs, these systems leverage the tumor microenvironment to achieve synergistic photodynamic–chemotherapeutic effects [164]. This reflects a growing emphasis on microenvironment-responsive and theranostic integration. Another important trend involves NIR (near-infrared spectroscopy)-responsive COFs that unify PDT, photothermal therapy (PTT), and hypoxia-activated chemotherapy under single-wavelength excitation [165]. By incorporating tumor-targeting ligands and stimuli-responsive drug release mechanisms, these platforms enhance tumor selectivity while maintaining therapeutic potency under hypoxic conditions. The convergence of multiple treatment modalities into one structurally programmable framework underscores the versatility of reticular materials in precision oncology. Beyond cytotoxic therapies, MOF-based systems are increasingly being engineered to modulate antitumor immunity. Manganese-containing MOFs enabling co-delivery of catalytic ribozymes and immune-activating metal ions demonstrate how gene silencing can be coupled with innate immune activation [e.g., cGAS–STING (cyclic GMP-AMP synthase–stimulator of interferon genes signaling) pathway stimulation]. Similarly, ROS-responsive MOFs that covalently integrate immune agonists allow light-controlled immunomodulator release synchronized with PDT-induced ICD [166]. These designs spatially and temporally coordinate tumor destruction with immune activation, amplifying systemic antitumor responses while minimizing systemic toxicity.

### Antibacterial

Antimicrobial resistance (AMR) has severely reduced the effectiveness of conventional antibiotics [167], making alternative approaches urgently needed. Compounding this problem, bacteria in infection sites often form biofilms, which create hypoxic microenvironments that weaken O_2_-dependent type II PDT. Type I PDT, therefore, offers a practical advantage for antimicrobial therapy under these conditions. Unlike small-molecule PSs, organic frameworks provide a modular platform: Metal-node selection, ligand engineering, and heterostructure design enable multifunctional integration [168].

**Fig. 17.**
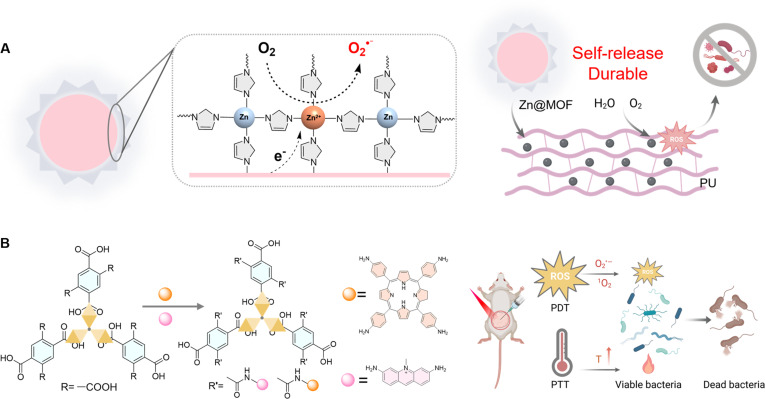
(A) O_2_ is reduced to O_2_^•−^ at the interface between the zinc core and the ZIF shell. Reproduced with permission of ref. [169], Copyright © 2023, American Chemical Society. (B) Schematic diagram of the synthesis of the PDT/PTT therapeutic agent HMIL-ACF-Por by post-synthetic modification. Reproduced with permission of ref. [172], Copyright © 2024, American Chemical Society. Figure created with Biorender.com.

MOFs have attracted strong interest as antibacterial materials. For instance, zinc-based core-shell MOF NPs (Zn@MOF) can spontaneously generate ROS upon exposure to air/moisture, producing O_2_^•−^ and H_2_O_2_ and killing a broad spectrum of microorganisms, including drug-resistant strains (Fig. 17A) [169]. When embedded in polyurethane matrices, the resulting composites retain durable antibacterial performance under repeated microbial challenge and aging. Mechanistically, the Zn core serves as an electron reservoir, feeding electrons to the MOF shell to form Zn^2+^ active centers; water and O_2_ diffuse through the porous shell to the interface, where Zn^2+^ promotes O_2_ reduction to O_2_^•−^. Zn@MOF also shows favorable biocompatibility and skin safety, supporting potential use in medical and consumer-care products [170]. Innovation-wise, this is an “always-on” ROS-generating architecture that does not rely strictly on high dissolved O_2_, which is valuable for biofilm-associated infections.

COFs can also serve as efficient antimicrobial photocatalysts. A COF PS (named as TPDA) was synthesized via Schiff-base condensation between TFP and 3,6-diaminoacridine (DAA) [171]. Enhanced conjugation and strong light harvesting from DAA give TPDA a narrow optical bandgap and broad absorption extending from UV to near-infrared. Upon irradiation, TPDA generates electron–hole pairs that transfer to O_2_, forming O_2_^•−^. ROS produced at the bacterial surface disrupt membranes and degrade intracellular proteins/DNA, leading to cell death. TPDA shows potent activity against both Gram-negative and Gram-positive bacteria under brief light exposure and can protect fish from skin infections. This demonstrates an innovation theme central to framework PSs: using ordered architectures to combine broadband light harvesting with efficient interfacial ROS generation. Because many molecular PSs suffer from poor water solubility and aggregation (π–π stacking), molecular-level dispersion within frameworks is an effective way to suppress aggregation and enhance photodynamic output. Li et al. [172] developed a multifunctional MOF-based PDT/PTT agent, HMIL-121–acriflavine–porphyrin (HMIL-ACF-Por), for antibacterial therapy. Under 808-nm NIR irradiation, HMIL-ACF-Por generates abundant ROS (O_2_^•−^ and ^1^O_2_) and photothermal heat (Fig. 17B), showing strong antibacterial activity against *Escherichia coli* and *Staphylococcus aureus* in vitro. The PDT-PTT synergy further reduces infection-associated inflammation and accelerates wound healing in *S. aureus* infection models.

## Conclusion and Prospect

Over the past decade, PDT, particularly type I PDT, has advanced rapidly and shown growing promise for treating malignant tumors. Among emerging type I PSs, organic framework materials stand out because of their programmable architectures, high porosity, and efficient light-triggered charge separation and electron transfer. These features make them attractive type I PS platforms that can mitigate the O_2_ limitation of conventional PDT and thus better suit hypoxic tumor microenvironments. Despite encouraging progress, generalizable, mechanism-guided design rules for high-performance type I organic framework PSs remain insufficient. This gap highlights the need for rational, structure-driven strategies rather than empirical optimization.

In this review, we summarize recent state-of-the-art advances that push organic framework materials toward practical type I PDT. We emphasize design–mechanism–function relationships, highlighting how controllable framework composition, topology, and electronic structure can be leveraged to regulate charge transfer pathways and ROS profiles. We aim to provide a design roadmap for developing next-generation type I PSs and to accelerate the translation of organic framework materials into clinically relevant phototherapeutic systems.

Although substantial progress has been made, most type I organic framework PSs remain at the proof-of-concept stage. Several key challenges must be addressed before organic framework-based PDT can achieve broad clinical translation:

1. Shifting the activation window without sacrificing type I reactivity. Most current type I organic frameworks are excited in the blue/visible region, where light penetration is intrinsically limited [173]. The next leap will require red-/NIR-addressable frameworks that preserve (or enhance) charge separation and radical generation. In practice, this means designing frameworks in which long-wavelength absorption, excited-state redox power, and electron transfer kinetics are co-optimized—rather than improved in isolation.

2. Engineering hypoxia resilience beyond “reduced O_2_ dependence”. Even for type I systems, O_2_ often remains involved in downstream radical chemistry; thus, “O_2_ independence” is frequently relative rather than absolute. A distinctive advantage of porous frameworks is that they can function as multimodal therapeutic scaffolds, enabling rational co-integration of PDT with complementary mechanisms (e.g., photothermal amplification, catalytic redox cycling, chemotherapy, or immunogenic cell death-oriented combinations). The innovation opportunity is to build single-platform, synergy-by-design systems that maintain efficacy across heterogeneous O_2_ landscapes.

3. Designing for clinical reality: biodegradation, clearance, and safety-by-construction. Translation will be determined less by proof-of-concept tumor ablation and more by pharmacology and toxicology. Many nanoscale frameworks face hepatobiliary clearance and potential long-term retention. A clear innovation direction is degradable, excretable framework engineering, using biodegradable linkers/building blocks, triggerable disassembly, and size/fragment control, to generate renal-clearable or otherwise efficiently clearable products while maintaining photochemical performance in vivo.

4. The in vivo behavior of framework-based PSs remains incompletely understood, as their size, morphology, surface properties, and composition collectively determine biodistribution, tumor accumulation, and clearance pathways. Although NPs in the 10- to 200-nm range can accumulate in tumors via the enhanced permeability and retention effect, they are typically cleared through the hepatobiliary system, potentially leading to prolonged hepatic retention [174]. To optimize pharmacokinetics and minimize off-target toxicity, strategies such as ultrasmall particle design, PEGylation, and the incorporation of renal-clearable motifs have been proposed.

5. Formulation design should be aligned with the intended clinical route of administration. Intravenous formulations require robust colloidal stability and compatibility with standard infusion systems to ensure safe and effective systemic delivery. In contrast, topical applications (e.g., for actinic keratosis or skin cancer) can be integrated into established dermatological PDT workflows using broadband or daylight activation, thereby enhancing clinical feasibility [175].

6. For successful clinical translation, framework-based PSs must comply with regulatory requirements including Good Manufacturing Practice (GMP) production, batch-to-batch consistency, sterility assurance, and comprehensive toxicological evaluation. Integrating technical development with regulatory planning from the design stage, together with early engagement with agencies such as the Food and Drug Administration (FDA) or European Medicines Agency (EMA), will help reduce translational risk and facilitate clinical implementation. To enhance clinical feasibility, future design strategies may align material properties with existing medical infrastructure. For example, since 630- and 690-nm laser systems are widely used in clinical PDT, the optical properties of framework materials could be rationally tuned—by modulating the D–π–A architecture of COFs or adjusting the ligand conjugation degree in MOFs—to precisely match these established excitation wavelengths [176]. Such spectral optimization would enable direct integration into current clinical workflows without the need for new light-source development. For dermatological applications, these materials could be incorporated into hydrogels or microneedle patches, making them compatible with existing topical administration and light-activation protocols (e.g., daylight or lamp-based PDT) [177]. Furthermore, integrating clinically approved imaging components—such as magnetic resonance imaging (MRI) contrast agents (e.g., Mn^2+^ and Gd^3+^) or PET isotope chelators—into the framework structure would allow real-time monitoring of tumor accumulation using standard imaging modalities [178]. This approach would enable precise determination of the optimal irradiation window and support personalized treatment planning.

7. Moving from empirical discovery to predictive, mechanism-guided screening. The field still lacks broadly applicable structure–activity relationships that can predict type I outcomes from scaffold design, which perpetuates trial-and-error synthesis. The next stage should couple quantitative mechanistic descriptors (e.g., excited-state lifetimes, charge separation yield, redox potentials, electron/hole mobility, and interfacial reaction rates) with data-driven discovery [including AI/ML (artificial intelligence/machine learning)] to establish predictive design maps for type I ROS profiles and therapeutic indices.

8. Enabling true theranostics: brighter imaging without blunting radical chemistry. Many organic frameworks exhibit modest fluorescence quantum yields, limiting high-fidelity imaging and real-time treatment guidance. The challenge is not simply to increase brightness, but to do so without diverting excited-state pathways away from electron transfer. A key frontier is orthogonal photophysics engineering—separating imaging and therapeutic channels (spatially, energetically, or temporally) to achieve robust imaging, accurate dosimetry, and type I ROS output within a single platform.
